# Genome-wide investigation of outbreak-associated *Vibrio cholerae* in Gujarat, India identifies antimicrobial resistance genes, virulence determinants, and mobile genetic elements

**DOI:** 10.3389/fmicb.2026.1851551

**Published:** 2026-07-02

**Authors:** Minal Bhure, Nitin Shukla, Harshal Purohit, Nimesh Patel, Priyank Chavda, Madhulika Mistry, Hitesh Shingala, Bhavin Solanki, Chirag Shah, Madhvi Joshi, Chaitanya Joshi, Snehal Bagatharia, Ramesh Pandit

**Affiliations:** 1 Gujarat Biotechnology Research Centre, Department of Science and Technology, Government of Gujarat, Gandhinagar, Gujarat, India; 2 Pandit Deendayal Upadhyay Medical College, Civil Hospital campus, Rajkot, Gujarat, India; 3 M.P. Shah Govt. Medical College, Bedi Road, Pandit Nehru Marg, Indradeep Society, Jamnagar, Gujarat, India; 4 Ahmedabad Municipal Corporation (AMC), Health Department, Sardar Patel Bhavan, Danapith, Ahmedabad, Gujarat, India; 5 Ahmedabad Municipal Corporation (AMC), Health Department, VBDC Branch, Aarogya Bhavan, Ahmedabad, Gujarat, India; 6 Department of Veterinary Biotechnology, College of Veterinary Science and Animal Husbandry, Kamdhenu University, Anand, Gujarat, India; 7 Hester Biosciences Limited, Meda-Adraj, Kadi, Mehsana, Gujarat, India

**Keywords:** antimicrobial resistance, cholera, integrative conjugative elements, phylogenomic, *Vibrio cholerae*, wastewater surveillance

## Abstract

This study investigates the 2024 cholera outbreak in Gujarat, India, utilizing combined whole-genome analysis of clinical *Vibrio cholerae* isolates and wastewater surveillance. A total of, 69 *V. cholerae* isolates were recovered from affected patients, predominantly belonging to the O1 serogroup (51 isolates). Antimicrobial susceptibility test (AST) of 34 isolates revealed complete resistance to ampicillin and partial resistance to cotrimoxazole, whereas all isolates were susceptible to doxycycline, ciprofloxacin, chloramphenicol, tetracycline, and gentamicin. Whole-genome sequencing of 20 selected isolates revealed that the isolates belong to the seventh pandemic El Tor (7PET) lineage, sequence type ST69. Phylogenomic analyses using a multi-method approach, core genes, Composition Vector (CV) Tree, SNPs, and multilocus sequence typing (MLST) showed tight clustering with limited diversity among the isolates. All isolates contained 13–15 antimicrobial resistance genes, with high consistency between genotype–phenotype for most antibiotics, although discordance was observed for ciprofloxacin, cotrimoxazole, and chloramphenicol. Sixteen genes were identified as virulence factors, and 11 isolates also had *ctxA/ctxB*. All isolates also had two to four integrative conjugative elements (ICEs) containing antimicrobial resistance genes (ARGs) and important *Vibrio cholerae* pathogenicity islands (VPI-1, VPI-2) and *Vibrio cholerae* seventh pandemic islands (VSP-1, VSP-2). The pangenome analysis highlights extensive genomic flexibility within species, likely driven by horizontal gene transfer and ecological adaptation; however, further outbreak-specific investigations are required to determine their direct role in current outbreak. The detection of *ctxA*-positive signals in wastewater, 20% (28/140) of the samples, suggests a possible surveillance signal during the outbreak. These results highlight the presence of antimicrobial-resistant 7PET O1 El Tor strains in Gujarat outbreaks and support continued genomic monitoring to guide focused public health interventions in endemic areas. Furthermore, this study also underscores the importance of wastewater surveillance for monitoring *V. cholerae*.

## 1 Introduction

*Vibrio cholerae*, a Gram-negative, facultative anaerobic, curved, highly motile bacterium with a monotrichous flagellum, is the causative agent of for cholera disease. This infectious agent remains a significant global public health concern, particularly in regions with poor water sanitation and hygiene infrastructure. Cholera is a severe and potentially fatal gastrointestinal disease that causes acute watery diarrhoea. The first recorded cholera pandemic had occurred during the 19th century, and since then, six pandemics have caused millions of deaths worldwide. The current seventh pandemic, which began in 1961 in South Asia, is still infecting people all over the globe (WHO). Robert Koch isolated *V. cholerae* for the first time in pure culture from the samples collected from the cholera pandemic in 1817–1824 at Kolkata, India (Lippi and Gotuzzo, 2014; Donatella et al., 2016). The disease then spread from the Bengal region to Southeast Asia, the Middle East, Europe, and Eastern Africa through trade routes. India is considered a hotspot for cholera outbreaks and witnessed 68 and 559 outbreaks during 1997–2009 and 2009–2017, respectively (Muzembo et al., 2022).

The pathogen is classified into more than 200 serogroups; however, cholera outbreaks are mainly caused by two serogroups: O1 and O139 (Finkelstein, 1996; Montero et al., 2023; Sabir et al., 2023). *V. cholerae* O1 is responsible for all historical and recent pandemics, whereas *V. cholerae* O139 caused sporadic outbreaks in some Asian regions (Faruque et al., 2003; Parvin et al., 2021; Ramamurthy et al., 2022). The illnesses caused by the two serogroups are identical; however, prior immunity to *V. cholerae* O1 El Tor does not appear to protect against illness caused by *V. cholerae* O139 (Morris, 1995; Albert, 1996). The seventh cholera pandemic, caused by the El Tor biotype of *V. cholerae* O1, has exhibited ongoing genetic diversification, leading to the emergence of new variants with altered virulence and antibiotic resistance profiles (Domman et al., 2017; Montero et al., 2023). Key genomic features of *V. cholerae* include the presence of the cholera toxin (CTX) encoded by the *ctxAB* genes and other virulence factors such as the toxin-coregulated pilus (tcpA), which facilitates intestinal colonization (Ramamurthy et al., 2019; Montero et al., 2023). Additionally, genomic studies have revealed the increasing prevalence of antimicrobial resistance genes, often carried on mobile genetic elements such as integrative conjugative elements (ICEs) and plasmids, raising concerns about treatment efficacy and outbreak management (De, 2021; Lassalle et al., 2023).

Genome sequencing has revolutionised the research in microbiology, pathogen biology, and disease outbreak characterisation, enabling rapid characterisation of bacterial pathogens. This approach has been valuable in tracing the origin and spread of *V. cholerae* during previous outbreaks (Mutreja et al., 2011; Hasan et al., 2012). Cholera outbreaks continue to pose serious public health threats, with recent incidences highlighting the need for genomic surveillance to track pathogen evolution, transmission dynamics, virulence determinants, antimicrobial resistance patterns, and the presence of mobile genetic elements (Mohiuddin et al., 2023; Monir et al., 2023; Luo et al., 2024). Furthermore, to aid prevention and hotspot control, in 2017, the WHO launched a global initiative (Global Task Force on Cholera Control, GTFCC) to eliminate cholera as a public health threat by 2030 (Legros, 2018; Muzembo et al., 2022). Besides, in the current time, wastewater surveillance has also become an important method in tracking certain pathogens, including *V. cholerae* (Chigwechokha et al., 2024; Manna et al., 2024). This approach provides an early warning indication and insight into community health (Kumar et al., 2021a). In this context, insights into the genomes of *V. cholerae* isolated from the ongoing outbreaks, its antimicrobial resistance pattern and genes, presence of mobile genetic elements and virulence determinants will be very crucial to control this pathogen.

Therefore, this study aimed to characterize and understand the genetic diversity, virulence determinants, antimicrobial resistance and associated genes, and mobile genetic elements in *V. cholerae* strains isolated during the outbreak. Here, we present a comprehensive genomic analysis of 20 *V. cholerae* isolates from an outbreak in Gujarat, India. We also track the presence of the causative agent in the water and wastewater samples collected during the same period.

## 2 Materials and methods

### 2.1 Sample collection

During June to October 2024, a sudden rise in the cases of cholera was reported from different districts of Gujarat state, India, with June–August was the peak period. These cases were mainly reported from Jamnagar, Rajkot, Ahmedabad, Gandhinagar, and Anand districts, with Rajkot and Jamnagar being the most affected areas. Hospitals in these areas were instructed by the Health and Family Welfare Department, Government of Gujarat, to send the cultures of *V. cholerae* to Gujarat Biotechnology Research Centre (GBRC) for whole-genome analysis. These isolates were recovered by the hospitals from the patients’ stool samples. As a result, a total of 69 cultures of *V. cholerae* were received for detailed genomic analysis which were isolated during June–August 2024. Along with the cultures, some hospitals also sent antimicrobial susceptibility test (AST) data for the isolates, though not for all of them, because AST testing was performed independently by different hospitals. At the same time, the State Government also instructed municipal corporations in the affected areas to send samples of water and wastewater to GBRC for the detection of *V. cholerae*. As a result, a total of 140 samples of wastewater from Rajkot and Jamnagar, and 17 drinking water samples Ahmedabad city were received for the analysis.

### 2.2 DNA extraction and whole genome sequencing of clinical isolates

For whole genome sequencing, 20 isolates were randomly selected while ensuring representation across different districts. The selected isolates originated from Ahmedabad, Jamnagar, and Rajkot, the most affected cities. These are the same three cities from where the water and wastewater samples were also received. The selected isolates were representative of the outbreak in terms of geographic origin, serogroups, serotypes, and AST profiles. Out of these 20 isolates, AST results were available for 15 isolates. For cotrimoxazole, 3/15 were susceptible, while the remaining 12 were resistant. For 10 isolates, serotyping data was also provided by the hospitals, based on which all 10 belong to *V. cholerae* O1, in that two were Ogawa and three were Inaba (Supplementary Table S1).

From the isolates, total DNA was extracted using QIAamp DNA Mini Kit (Qiagen, Hilden, Germany). Then, QIAseq FX DNA Library Kit (Qiagen, Germany) was used to prepare whole-genome shotgun libraries. The quality of libraries was evaluated on LabChip GX Touch nucleic acid analyzer using NGS 3k chip (Revvity, MA, US), and concentrations were measured in Qubit 4 Fluorometer using Qubit™ 1X dsDNA High Sensitivity (Thermo Fisher Scientific, US). All libraries were sequenced on an Illumina sequencer with 500 cycles, 2 × 250 bp paired-end chemistry with targeted sequencing depth of > 50x coverage.

### 2.3 Genome assembly and its quality assessment

The Illumina DRAGEN v4.0.3 server was used to demultiplex the generated data, along with the removal of adapter sequences. Low-quality reads were filtered using a minimum quality threshold of Q30 using PRINSEQ++ v1.2 (Cantu et al., 2019). Quality of reads was assessed before and after trimming using FastQC v0.11.9. The clean reads were assembled to generate a *de novo* assembly using Unicycler v0.5.0 with a minimum contig length of 300 bp (Wick et al., 2017). The quality of assemblies was evaluated using QUAST v5.0.2, while CheckM (Parks et al., 2015) and BUSCO (Simão et al., 2015; Manni et al., 2021) were used to assess the contamination and completeness of the genome, respectively. All assembled genomes were uploaded to PubMLST (Jolley et al., 2018) and Type (Strain) Genome Server (TYGS) (Meier-Kolthoff and Göker, 2019) for multilocus sequence typing, whole genome digital DNA–DNA hybridization, and 16S-based identification. Strain typing (cgMLST) was performed against all loci of *V. cholerae* available at the PubMLST server. Unless otherwise specified, all bioinformatic analyses were performed using default parameters.

### 2.4 Analysis of genomic features

#### 2.4.1 Analysis of antimicrobial resistance genes, virulence factors, and mobile genetic elements

Full annotation of all the assembled genomes was performed using the comprehensive genome analysis module available at the BV-BRC (PATRIC) server (Shukla M. et al., 2026), where antimicrobial resistance genes (ARGs) were identified and annotated using the Comprehensive Antibiotic Resistance Database (CARD) (McArthur et al., 2013), and virulence factors (VFs) were identified using the Virulence Factor Database (VFDB) (Zhou et al., 2025). Antimicrobial resistance genes (ARGs) carrying mobile genetic elements (MGEs) were identified using VRprofile2 (Wang et al., 2022). Pathogenwatch was used to check the pathogenicity and identification of AMR and virulence genes (Argimón et al., 2021). Additionally, fIDBAC (Liang et al., 2021) was also used to screen ARGs/VFs genes. The presence of any CRISPR sequences was identified using CRISPRCasFinder (Couvin et al., 2018). Integrative and conjugative elements (ICE) were detected using ICEfinder ICEberg3.0 (Wang et al., 2024). BLAST Ring Image Generator (BRIG) was used to compare all 20 genomes with reference genome *V. cholerae El Tor N1696*1, and custom annotation for genomic islands comprising genes was done (Alikhan et al., 2011).

#### 2.4.2 Phylogenetic analysis

Protein-based phylogenetic analysis of all the assembled genomes was performed using Composition Vector Tree Version 4 (CVTree4 v4.0), (Qi et al., 2004; Zuo, 2021) with all available 893 genomes of *V. cholerae*, and *Vibrio parahaemolyticus RIMD 2210633* was used as an outgroup. It performs Composition vector (alignment-free) sequence comparison and calculates k-mer frequency–based distance using Neighbor-Joining method. These 893 genomes were isolated from 40 different countries. Furthermore, a phylogenetic tree was generated from the core genes using the PGAP2 pipeline (v2.2.0) and FastTree, which by default use 1,000 bootstraps to create approximately-maximum-likelihood phylogenetic trees. The resulting trees was visualised with iTOL (v7.5) and were annotated with the label of the country and the year of isolation (Letunic and Bork, 2024). The details of *V. cholerae* genomes used for phylogeny are provided in Supplementary Table S2. To resolve the dissimilarities among these 20 isolates of this current outbreak, we further performed SNP-based phylogeny using the fIDBAC online platform (Liang et al., 2021) with default settings. For SNP-based phylogeny, fIDBAC uses MUMmer for whole genome alignment, find SNPs among the genomes, and build a tree. autoMLST2.0 was also used to build a phylogeny based on MLST genes (Pourmohsenin et al., 2025).

#### 2.4.3 Pangenome analysis

A species-level pan-genome analysis was conducted using 893 *V. cholerae* genomes, which were used for CVtree phylogeny to maintain consistency in the analysis. All these 893 genomes were downloaded from NCBI via the NCBI Datasets package (v17.3.0). To these, 20 outbreak sequences from this study were combined, resulting in a total of 913 genomes. All 913 genomes were re-annotated using Prokka (v1.15.6). The resulting GFF files were used for analysis, which included the identification of core and accessory genes, as well as the construction of a pan-genome matrix using PGAP2 (v2.0). Here ‘strict core’ genes are those which are present in all (100%) genomes; ‘core’ gene are those found in ~99% genomes, ‘soft-core’ represent genes present in majority (~95–99%) of the genomes, ‘shell’ stand for those genes which are found in variably across the genomes examines (~15–95%) and ‘cloud’ is for rare genes, present in <15% of genomes (Bu et al., 2025; CD Genomics, 2025).

### 2.5 Detection of *Vibrio cholerae* from water and wastewater samples

From each of the water and wastewater samples, 50 mL of sample was centrifuged at 10000 g for 15 min at 4 °C. The resulting pellet was used for total DNA extraction using the DNeasy Power Water Kit (Qiagen, Germany). To detect the presence of *V. cholerae*, we used species-specific primers, targeting the *ctxA* gene, forward 5′ GCAGTCAGGTGGTCTTATGC 3′ and reverse 5′ CGTGCCTAACAAATCCCGTC 3′, developed by Hoshino et al. (1998). For PCR, 10 ng DNA from each sample was amplified using 5 μM each primer (forward and reverse) using EmeraldAmp GT PCR Master Mix (Takara Bio, USA) with the following thermal cycling conditions. Initial denaturation was carried out at 95 °C for 2 min, followed by 25 cycles of denaturation at 95 °C for 1 min, primer annealing at 58 °C for 30 s and extension at 72 °C for 1 min. Then, a final extension was carried out at 72 °C for 10 min. The amplified PCR products were run on 1.5% agarose gel. The samples showed a band near 116 bp were considered as a positive for the presence of *V. cholerae*.

## 3 Results

### 3.1 Serogroup, serotypes, and antimicrobial susceptibility

A total of 69 *V. cholerae* isolates were recovered by the hospitals from stool samples of patients with acute diarrhoea. At the serogroup level, 51 isolates were identified as O1, which is responsible for all historical and ongoing cholera pandemics. Moreover, 8 and 14 were further classified as Ogawa and Inaba serotypes, respectively. AST results are for 34 isolates were provided by the hospitals. All these 34 isolates showed almost similar pattern, resistant to ampicillin and susceptible to doxycycline, ciprofloxacin, chloramphenicol, tetracycline, and gentamicin. For cotrimoxazole, 29 isolates were resistant, while the remaining five were susceptible (Supplementary Table S3).

### 3.2 Whole genome-based characterization

#### 3.2.1 Genomic characteristics

The sequencing using Illumina platforms generated a total of 11.29 million reads (5.64Gb) with an average of 0.56 million reads (280 Mb) per sample (Supplementary Table S4). The details of assembly statistics are provided in Table 1. All the isolates were identified as *V. cholerae* using a multi-method approach, PubMLST and TYGS. The genome assembly size ranges from 3.9 to 4.0 Mb with an average N50 of 149.8 kb and L50 value 10. Based on the per sample data generated and assembled genome size, an average sequencing depth was approximately 70×. GC content of all the assembled genomes was ~47.58%. The average number of predicted coding sequences were 3,695 per isolate. All genomes are of high quality, having completeness 100% and contamination 0% except VP8 (completeness, 98.2%). This confirms the purity of the isolates as well as good laboratory practices being followed during the isolation to sequencing process. All assemblies are available at the Indian Biological Data Centre (IBDC) under the accession numbers provided in Table 1.

**Table 1:** Basic statistics of *de novo* assembled genomes of all 20 *Vibrio cholerae* isolates.

Sample ID	No. contigs	Total assembly length (Mbs)	Largest contig (Kbs)	N50	L50	GC (%)	Completeness	CDS	tRNA	rRNA	Hypothetical proteins	Proteins with functional assignments	INSDC accessions
IC1	75	4.02	632.1	197656	5	47.51	100	3,707	78	5	626	3,081	ERR15466559
IC2	87	3.99	496.7	134206	8	47.61	100	3,681	77	5	616	3,065	ERR15466560
IC3	78	4.01	627.4	235588	5	47.53	100	3,707	79	4	623	3,084	ERR15466561
IC4	86	4.01	501.9	166339	7	47.52	100	3,700	79	5	623	3,077	ERR15466563
IC5	81	4.01	569.5	235588	5	47.54	100	3,693	77	5	618	3,075	ERR15466564
IC6	73	4.01	663.6	235588	5	47.53	99.98	3,711	78	5	633	3,078	ERR15466565
IC7	89	4.00	559.7	197656	6	47.56	100	3,706	80	5	629	3,077	ERR15466566
IC8	77	4.02	571.2	235588	5	47.52	100	3,708	77	5	627	3,081	ERR15466567
IC9	74	4.01	497.0	171138	7	47.56	100	3,703	76	5	623	3,080	ERR15466568
IC10	107	4.01	501.9	129579	10	47.53	100	3,715	79	4	634	3,081	ERR15466570
VP1	202	3.94	110.6	38354	31	47.75	100	3,660	66	5	600	3,060	ERR15466571
VP2	111	3.99	337.3	110689	12	47.6	100	3,687	67	5	616	3,071	ERR15466572
VP3	132	3.98	433.8	109599	10	47.64	100	3,681	76	5	617	3,064	ERR15466573
VP4	119	4.00	446.7	134207	10	47.57	100	3,704	77	5	626	3,078	ERR15466574
VP5	113	3.99	283.7	121975	10	47.6	100	3,694	76	5	623	3,071	ERR15466575
VP6	124	4.00	204.1	95465	14	47.6	100	3,696	77	5	617	3,079	ERR15466576
VP7	122	3.99	272.2	107006	12	47.59	100	3,689	77	5	613	3,076	ERR15466577
VP8	132	3.96	247.1	101690	14	47.61	98.62	3,658	75	5	615	3,043	ERR15466578
VP9	130	3.99	272.2	110943	13	47.61	100	3,700	77	5	618	3,082	ERR15466579
VP10	110	4.00	288.9	127212	11	47.58	100	3,697	76	5	624	3,073	ERR15466580

Genomes were assembled using Unicycle and annotated using PATRIC server.

#### 3.2.2 Antimicrobial resistance gene profile

**Figure 1:**
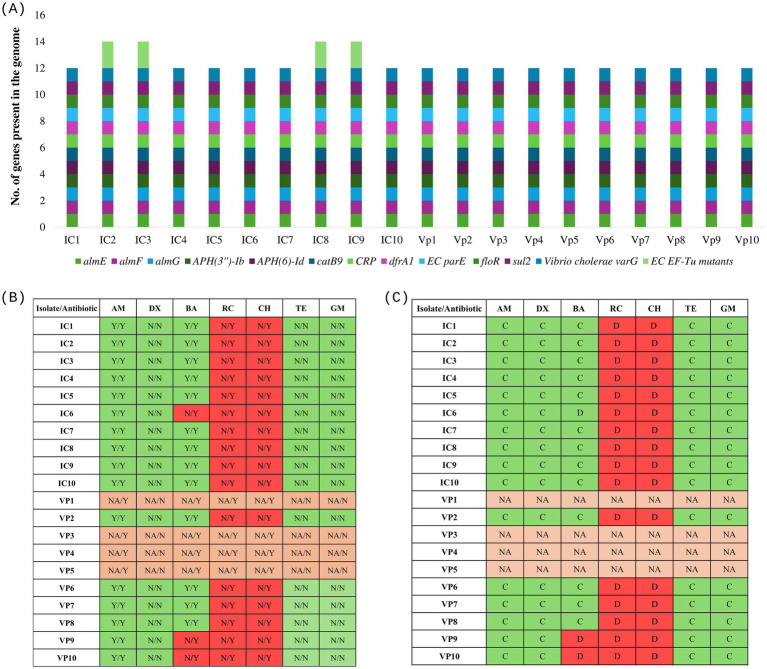
Antimicrobial resistance analysis of 20 *Vibrio cholerae* isolates. **(A)** The distribution of antibiotic resistance genes (ARGs) identified through whole-genome sequencing, **(B)** comparison of ARGs with phenotypic antimicrobial susceptibility testing (AST), and **(C)** concordance between phenotype and genotype data. In panel **B**, if an isolate is resistant to a particular antibiotic, then its phenotype was noted as ‘Y’, if it is susceptible, then was noted as ‘N’ under phenotype (P). Similarly, if a gene which confers resistance to an antibiotic is present in the genome, then ‘Y’ under Genotype (G) and inverse is noted as ‘N’, while “NA” indicates AST data was not available. Here data presented as phenotype ‘P’ and genotype ‘G’. In panel **C**, concordance ‘C’ indicates agreement (Y/Y or N/N), and discordance ‘D’ indicates mismatch between two methods.

Screening of ARGs using the CARD database revealed the presence of 13 antimicrobial resistance genes across all isolates (Figure 1A; Supplementary Table S5). The identified genes include *almE*, *almF*, *almG*, *APH(3″)-Ib*, *APH(6)-Id*, *catB9*, *CRP*, *dfrA1*, *Escherichia coli parE* conferring resistance to fluoroquinolones, and *floR*, *sul2*, *Vibrio cholerae* var*G*, and *Escherichia coli EF-Tu mutants* conferring resistance to pulvomycin. The details of antimicrobial-resistant genes, their resistance mechanism, antibiotics target, and the class for which the gene conferring resistance is provided in Supplementary Table S6. Among these, *Escherichia coli EF-Tu mutants* conferring resistance to pulvomycin was present in four isolates with duplicate copies and having the R234F mutation. All other genes are common across all 20 isolates, where *Escherichia coli parE* conferring resistance to fluoroquinolones with a D476N mutation, was noted. AMR prediction from Pathogenwatch revealed the presence of 15 antimicrobial resistance determinants for ampicillin, ceftazidime, carbapenems, broad-spectrum cephalosporins, chloramphenicol, ciprofloxacin, ceftriaxone, cefepime, furazolidone, nalidixic acid, nitrofurantoin, sulfamethoxazole, sulfisoxazole, streptomycin, and trimethoprim in all isolates. No possible genes conferring resistance to azithromycin, doxycycline, meropenem, rifampicin, and tetracycline were detected (Supplementary Table S7).

The phenotype–genotype concordance was calculated using the results of AST and AMR genes identified through WGS analysis. The antibiotics examined include ampicillin, doxycycline, cotrimoxazole, ciprofloxacin, chloramphenicol, tetracycline and gentamicin. If an isolate is resistant to a particular antibiotic, then its phenotype was considered as ‘Y’, if it is susceptible, then considered as ‘N’ under category phenotype (P). Similarly, if a gene which confers resistance to an antibiotic is present in the genome, then ‘Y’ under category genotype (G) and inverse is written as ‘N’ (Supplementary Table S8). Isolates for which AST results are unavailable (VP1, VP3, VP4, VP5 and VP6) were presented as ‘NA’ (Figure 1B). Based on this information, the isolate being resistant to an antibiotic and having a corresponding gene in its genome, as well as an isolate being susceptible to particular antibiotic and absence of corresponding gene is denoted as concordance (C), and mismatched cases are denoted as discordance (D) (Figure 1C). Complete genotype–phenotype concordance was observed for ampicillin, doxycycline, tetracycline, and gentamicin, where phenotypic susceptibility or resistance patterns were consistent with the presence or absence of corresponding resistance determinants. Discordance was observed for ciprofloxacin and chloramphenicol, where isolates remained phenotypically susceptible despite the presence of resistance-associated determinants, including the parE D476N mutation and the *floR*/*catB9* genes. For cotrimoxazole, most isolates showed concordance between phenotype and genotype based on the presence of *dfrA1* and *sul2* genes; however, IC6, VP9, and VP10 remained phenotypically susceptible despite carrying these resistance determinants. Detailed isolate-wise phenotype, resistance mechanisms, and concordance/discordance patterns are provided in Supplementary Tables S3, S6, S8.

#### 3.2.3 Pathogenicity and virulence factors

Annotation using Virulence Factor Database (VFDB) identified 16 virulent genes. Among all these, 15 are related to virulence, intracellular survival and replication, cell-to-cell spread, stress, regulation of gene expression, modulate host immune response, and cellular metabolism were common in all isolates (Supplementary Table S9). The *tktA* gene was present only in one isolate, IC10. Based on CG-MLST results, all isolates belong to sequence type (ST) 69 and mismatches in the core genome loci or number of allele differences between sequence types range from 10 to 71, where 71 were in the VP1 isolate. Pathogenwatch also confirm that all the isolates belong to ST 69. Additionally, Pathogenwatch classified genomes into serogroups, where 14 isolates belong to the O1 serogroup (Supplementary Table S10). Pathogenwatch identified a total of 11 virulence determinants, *ace*, *hapA*, *hlyA*, *makA*, *nanH*, *ompU*, *rpoS*, *toxR*, *vasX*, Lux Operon, and MSHA pilus in all isolates. Interestingly, cholera toxin (CT) encoding genes, *ctxA* and *ctxB,* were present only in 11 isolates. *ChxA* and *stn* were absent in all the isolates (Supplementary Table S11). No CRISPR system was found in any of the isolates.

#### 3.2.4 Integrative and conjugative elements

**Figure 2:**
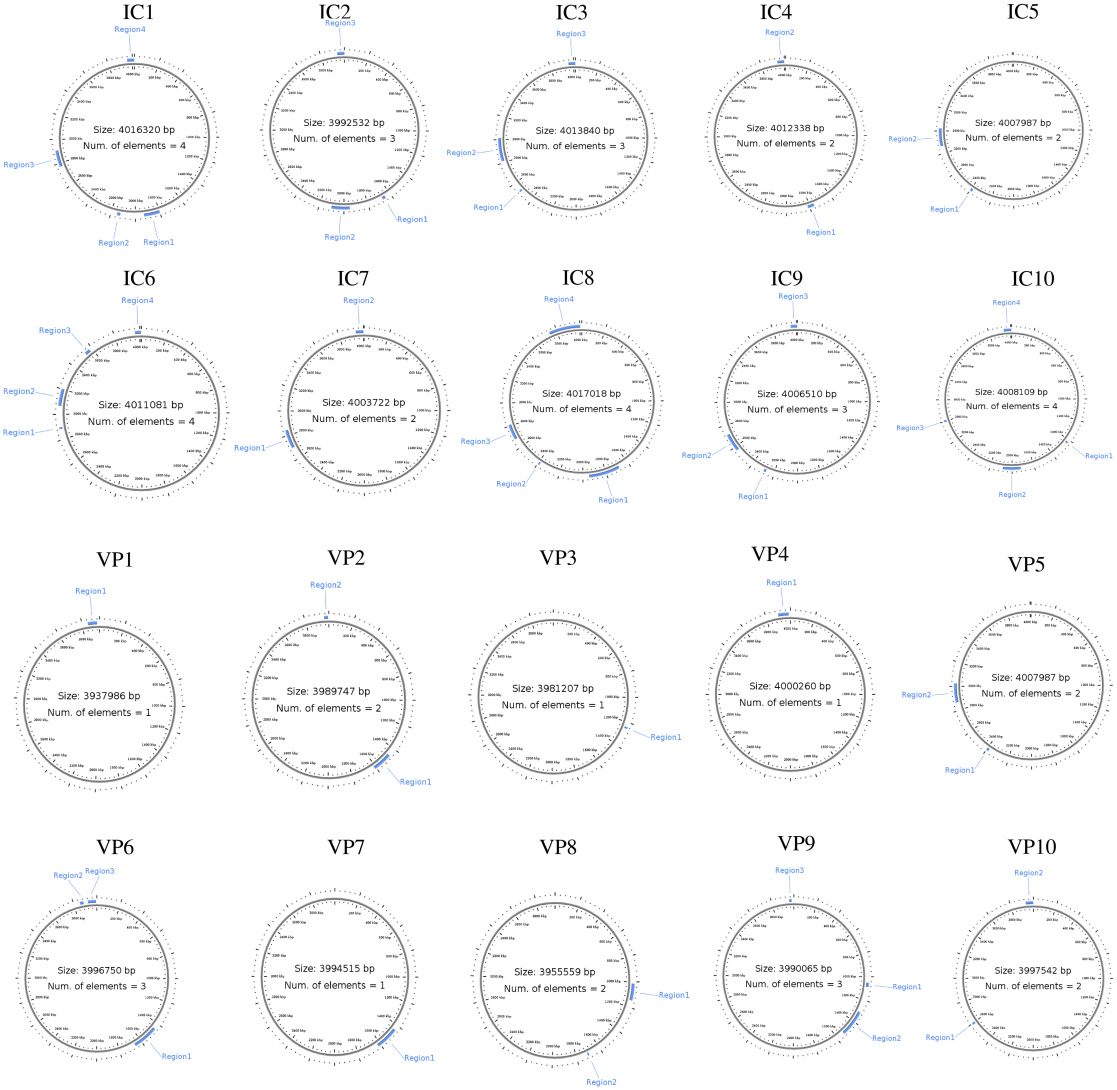
Distribution and localization of integrative and conjugative elements (ICE) in 20 *Vibrio cholerae* isolates. The presence of mobile genetic elements highlighted blue regions representing integrative and conjugative elements (ICEs). Mobile genetic elements were detected via ICEfinder (ICEberg 3.0).

Screening of mobile genetic elements revealed the presence of 2 to 3 regions in most of the isolates, while 4 regions were found in each IC1 and IC6 isolate (Figure 2). The identified antimicrobial resistance genes, virulence factors, and ARGs containing MGEs screened through VRProfile2 are provided in Supplementary Table S12. Analysis revealed the presence of transposase, integrase, OriT, antibiotic resistance, virulence factor, hypothetical protein, T4SS, T4CP, relaxase, SCCtype, SCCccr, and others (Supplementary Figure S1). This analysis also confirmed the presence of at least one ARG containing MGEs in all 20 isolates. Surprisingly, the *dfrA1* gene was found to be carried by an integron located within the chromosomal region in 19 *Vibrio* genomes, whereas in isolate VP1, it was in the plasmid region.

#### 3.2.5 Genomic islands

**Figure 3:**
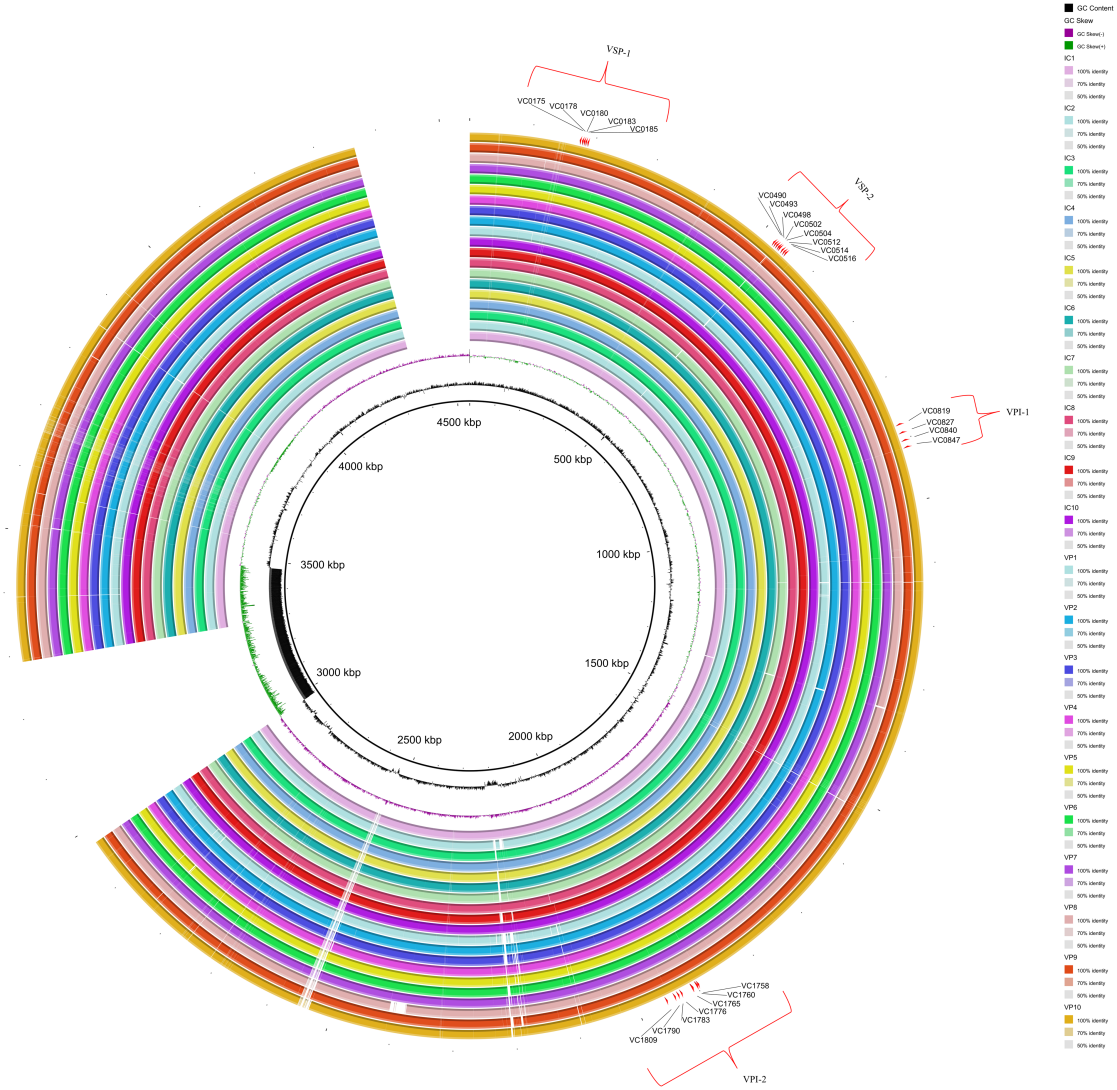
Comparative genomic analysis of *Vibrio cholerae* isolates highlighting conserved Genomic Islands (GIs). Genome comparison of 20 *V. cholerae* isolates was performed using the BLAST Ring Image Generator (BRIG), with *V. cholerae* El Tor N16961 as the reference genome. Concentric rings, color-coded by isolate, represent individual genomes aligned using BLAST, with colour intensity indicating sequence similarity. Annotated regions confirm the consistent localization of VSP-1, VSP-2, VPI-1, and VPI-2 across all isolates. The innermost rings depict GC content and GC skew, while gaps in the outer rings indicate localized sequence divergence or deletions relative to the reference.

We compared genomes of all 20 isolates with the reference genome *V. cholerae* N16961 and screened for the presence of genomic islands. A total of 4 genomic islands, *V. cholerae* pathogenicity island 1 (VPI-1), *V. cholerae* pathogenicity island 2 (VPI-2), *V. cholerae* seventh pandemic island 1 (VSP-1), and *V. cholerae* seventh pandemic island 2 (VSP-2) were identified. All 20 isolates possess these islands, while a few isolates lack some genes of it. IC7, VP1, VP6, VP7, VP8, VP9 and VP10 lack VC0847 (integrase, phage family) genes from VPI-1. Similarly, IC1, IC2, IC4, IC7 and all VP isolates except VP10 lack a few genes from VSP-2. While VSP-1 and VPI-2 islands harbour all genes across all isolates (Figure 3; Supplementary Table S13).

#### 3.2.6 Phylogeny analysis

**Figure 4:**
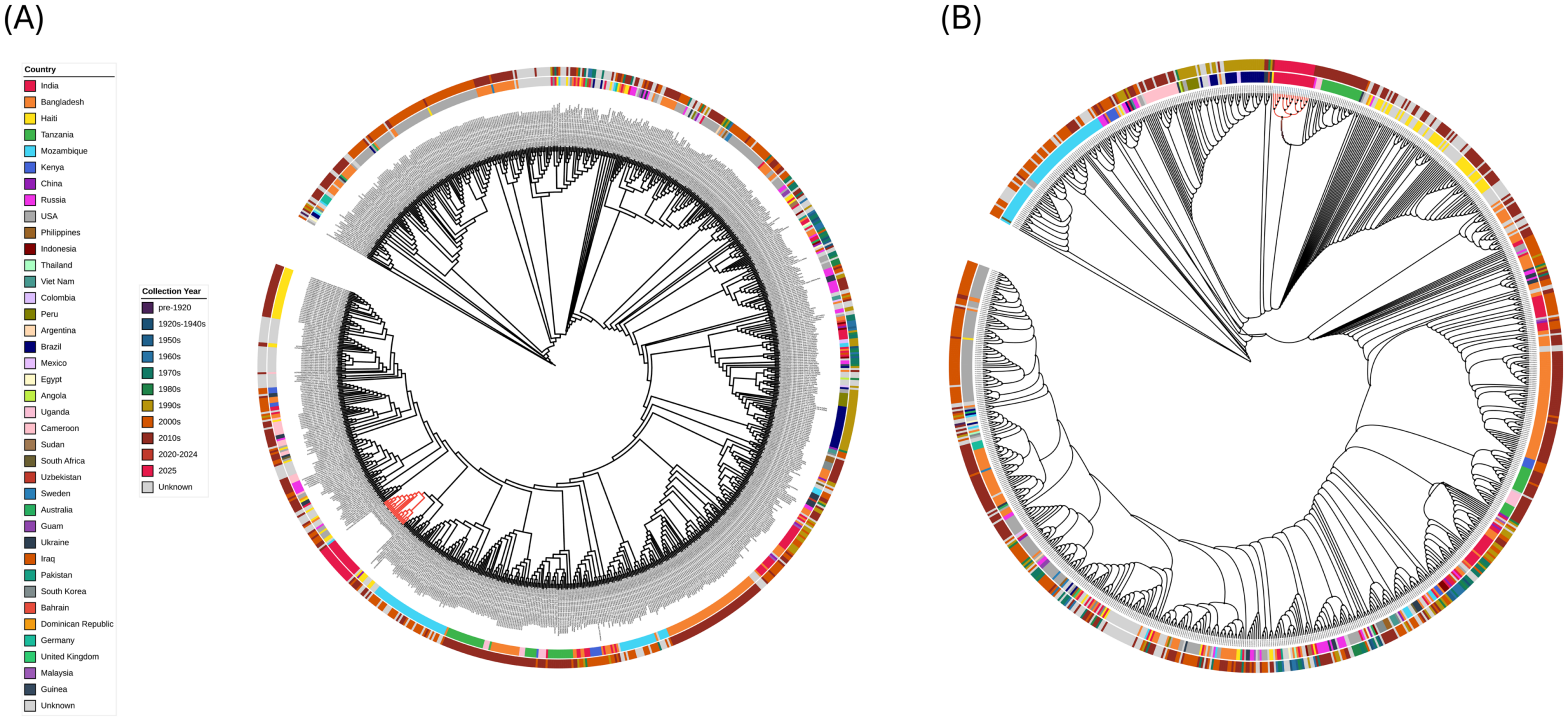
Phylogeny analysis of *Vibrio cholerae* isolates. **(A)** a protein-based phylogeny of 20 isolates from this study and 893 *V cholerae* isolates genomes available at CVTree v4.0. *Vibrio parahaemolyticus* RIMD2210633 was used as an outgroup. **(B)** A core genes-based phylogeny of 913 *V. cholerae* genomes using PGAP2 pipeline and FastTree. The trees were visualized using iTOL v7.5 and annotated by the country of origin (inner circle), and year of isolation (outer ring). Unavailability of information regarding country or year was annotated as ‘Unknown’.

CVTree is a composition-vector-based phylogenetic analysis tool that builds phylogenetic trees using k-mer frequency patterns from whole genome sequences. A protein-based phylogeny showed that all the 20 isolates formed a tight and separate clade, showing close relatedness to older strains isolated in year 2002, 2005, 2010, and 2015. These included four isolates from Haiti (two from 2010 and one each from 2012 and 2014), and one isolate from China collected in 2005. For two isolates, one each from the years 2002 and 2025, the source country information was unavailable (Figure 4A). The core gene-based phylogeny built using PGAP2 (v2.0) showed close relatedness to strains isolated in the year 2010 from Tanzania (21 isolates), Cameroon (one isolate), Russia (one isolate), and one from India. The Indian strain of the year 2010 was very close to the current isolates (Figure 4B). These results are comparable with that of CVtree-based phylogeny.

An average nucleotide identity (ANI-AAI matrix) analysis revea1ed 100% identity among the isolates (Supplementary Figure S2). Yet, according to the ANI-based phylogeny, VP1 stand out differently from the others (Supplementary Figure S3A). Therefore, to see the differences among these 20 isolates, we further performed SNP-based phylogenetic analysis, where VP1 also stands separate (Supplementary Figure S3B). Details of SNPs among the isolates are provide in Supplementary Table S14. Based on the multi-locus sequence-based phylogeny, VP7 and IC4 were clearly separated from the other isolates. The rest isolates formed two clades, one with VP9, VP6, IC3, VP1, VP4, IC1, IC2 and another with IC6, IC8, IC10, VP2, VP10, VP5, VP3, IC5, IC9, IC7, and VP8 (Supplementary Figure S3C). Further, it is to be noted that isolate VP1 has the highest number of SNPs when compared with other isolates. The VP1 isolate was from Rajkot city, while the remaining 19 were from samples from Ahmedabad and Jamnagar city.

#### 3.2.7 Pan-genome analysis

**Figure 5:**
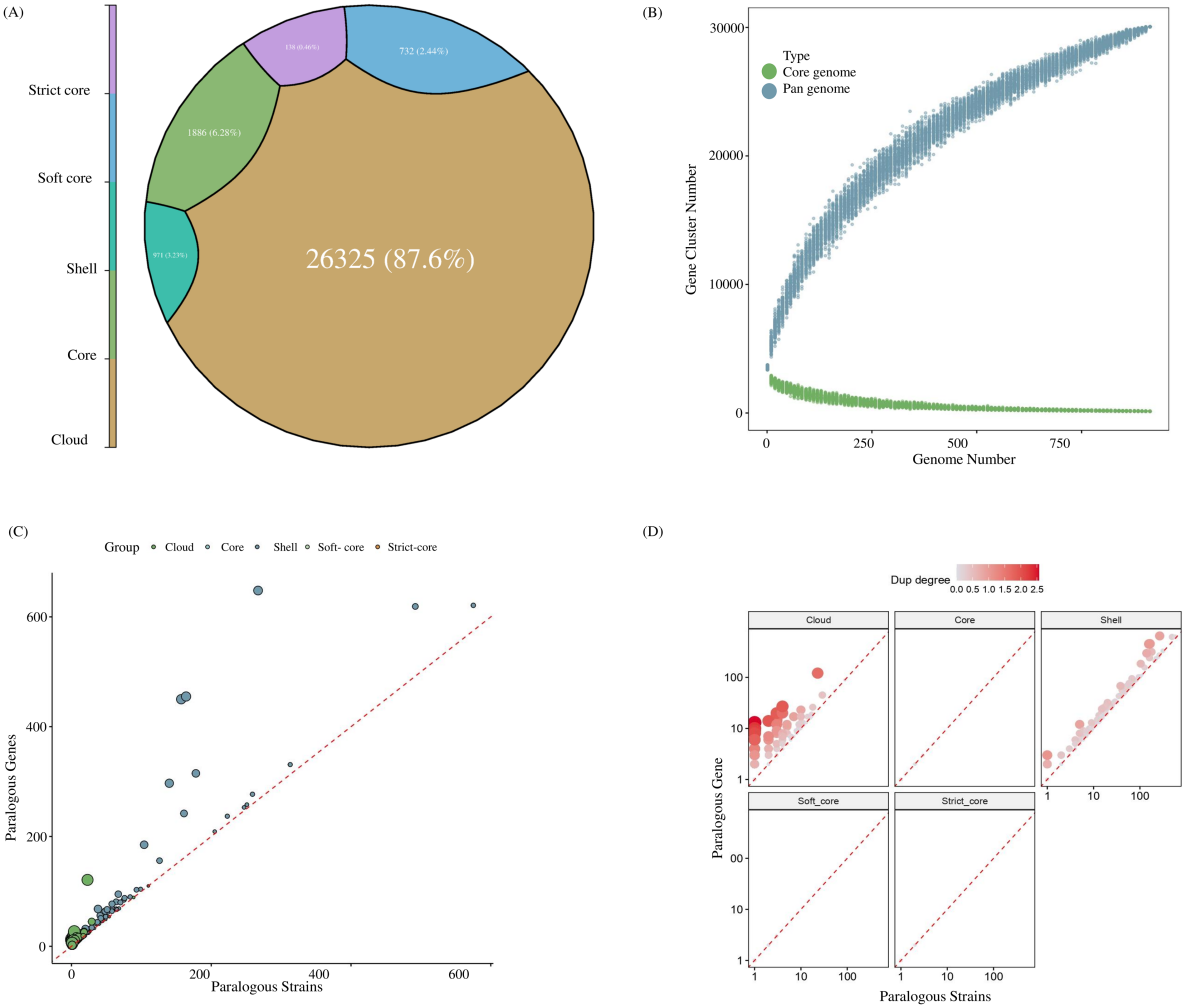
Pangenome and paralogous gene distribution of 913 *Vibrio cholerae* genomes. Pangenome profile of 913 *V. cholerae* generated using PGAP2 pipeline. **(A)** Numbers and proportion of each pan-group. **(B)** Rarefaction curve showing the pangenome profiles of gene cluster on y-axis and number of genomes on x-axis. **(C)** Number of paralogous genes in each cluster across different pan-groups for *Vibrio.* **(D)** Scatter plot describing number of paralogous genes in each cluster, with deeper red hues indicating a higher number of duplicated genes in a strain.

A species-level pangenome analysis using 913 *V. cholerae* genomes, including 20 from this study, revealed a highly diverse and open pangenome. The species-level pan-genome comprised 30,052 gene clusters, which include 1,886 (6.28%) core genes, 732 (2.44%) soft-core genes, 971 (3.23%) shell genes, 26,325 (87.6%) cloud genes, and 138 (0.46%) strict core genes (Figure 5A). With the addition of new strains, the pangenome rarefaction curve shows a steady rise in the total number of gene clusters (pan genome), reaching about 30,000 gene clusters without showing a plateau. In contrast, the core genome decreased rapidly initially and then levelled off at a much lower number as more genomes were included (Figure 5B). The distribution of paralogous genes among the cloud, shell, soft-core, and strict core pangenome groups revealed distinct differences in gene duplication patterns (Figure 5C). Most duplicate genes were found in the cloud and shell genomes; the core and soft-core genomes had fewer duplicate genes and less variation. Additionally, as the number of strains increased, an increasing trend in paralogous gene counts, particularly within the shell genome, was noted, suggesting strain-specific expansion. The faceted analysis also showed that the strict-core and soft-core genomes displayed limited duplication with lower variability, while the cloud and shell genomes had higher duplicated genes (Figure 5D). To check the outbreak-level genomic variation among the 20 Gujarat isolates, we performed Pangenome analysis among these isolates. The analysis showed that several genes were missing or were unique to some isolates, Supplementary Table S15. However, these results should be interpreted with caution, as missing genes in other assemblies may be of fragmented assemblies and true absence.

### 3.3 Presence of *Vibrio cholerae* in water and wastewater samples

Based on PCR results of the presence of the *ctxA* gene, out of the total 140 wastewater samples, 28 (20%) samples were found to be positive for the presence of *V. cholerae*. A representative image of PCR-Gel electrophoresis is provided as Supplementary Figure S4. None of the drinking water samples from Ahmedabad city was positive for the presence of *ctxA* gene. The reason may be that all the samples of Ahmedabad were drinking water, while those from Rajkot and Jamnagar were wastewater (Supplementary Table S16).

## 4 Discussion

Cholera outbreaks are a significant global health issue as repeated outbreaks have been reported globally. Whole-genome sequencing of *V. cholerae* continues to provide valuable insights into its genetic diversity, presence of antimicrobial resistant genes, and mobile genetic elements, highlighting the pathogen’s evolving threat. Recent research shows the importance of horizontal gene transfer, especially of the CTXφ prophage and SXT/R391-like integrative conjugative elements, for increasing virulence and AMR in *V. cholerae* (Kachwamba et al., 2017; Chaguza et al., 2024). Other studies found that *V. cholerae* had virulence genes, a mixed profile of antimicrobial resistance genes and mobile genetic elements. Studies have also reported a decrease in resistance to trimethoprim-sulfamethoxazole, however, there is an alarming increase in the resistance to azithromycin (Li et al., 2025). These data not only show the adaptability of *V. cholerae* but also highlight the alarming nature of their genetic plasticity. Thus, genomics-based research points up the need for ongoing global sequencing surveillance to monitor lineage shifts, AMR genes and mutations in them, virulence factors, and the presence of MGE, which will help guide focused public health measures.

A significant increase in cholera cases occurred in several Gujarati districts between June and October 2024. Two districts, Rajkot and Jamnagar, had the highest cases reported, with additional cases also reported in Ahmedabad, Gandhinagar, and Anand. The state officials implemented coordinated efforts for clinical and environmental monitoring in response to this concerning increase. The rise in cases happens at the time as the usual seasonal trend of cholera during the monsoon in western India, when flooding and heavy rainfall increase the risk of fecal-oral transmission through contaminated water (Matteo et al., 2014). This study looked at the genetic characteristics of 20 clinical *V. cholerae* isolates from the affected cities and screened the presence of pathogen in 157 water and wastewater samples. This research allowed us to look at the genomics of the *V. cholerae* pathogen, including the genes that make it resistant to antimicrobials, mobile genetic elements, and the virulence factors.

The genome analysis of *V. cholerae* shows that all isolates are related to the strains that caused the 7th cholera pandemic. The clinical isolates were mostly *V. cholerae* O1, which aligns with the global trend of O1 El Tor being the dominant strain in the ongoing seventh pandemic (Ramamurthy et al., 2022; Irenge et al., 2024). All isolates were resistant to ampicillin but mostly susceptible to doxycycline, ciprofloxacin, chloramphenicol, tetracycline, and gentamicin. This is like what has been seen in outbreaks in India (Samal et al., 2025) and other places across the globe (Liu et al., 2022; Yuan et al., 2022; Mageto et al., 2025) over the past 10 years, where resistance to ampicillin and cotrimoxazole has been quite common, while doxycycline and tetracycline still show effectiveness. Although recent reports suggest emergence of MDR *V. cholerae* (Hossain et al., 2025; Nair et al., 2025; Samal et al., 2025), the isolates investigated in this study are found to be AMR only, i.e., they were resistance to only two antibiotics out of seven tested. The resistance was observed towards β-lactams (ampicillin) and folate pathway inhibitors (cotrimoxazole). Whole-genome sequencing of 20 *V. cholerae* isolates provided important understandings of their genetic structure and virulence determinants. The analysis showed that these isolates are part of sequence type 69 (ST 69), a typical signature of the seventh pandemic El Tor (7PET). This strain has been prevalent in ongoing outbreaks across Asia and Africa (Irenge et al., 2020; Bhandari et al., 2021; Liam et al., 2021; Yun et al., 2023; Jaeyres et al., 2026). This finding is consistent with global phylogenomic studies that look at how *V. cholerae* spreads from one region to another (Nair et al., 2025). These studies have found that the seventh pandemic El Tor strain is linked to outbreaks in Haiti and that sequence type 69 is found in India (Ayyappan et al., 2024). This shows *V. cholerae* is still a problem, and we need to keep track of how it is spreading and which types are most common.

As mentioned earlier, the outbreak occurred during the monsoon season, a high-risk period for cholera in India and this time, cholera cases increased in several districts in Gujarat. However, phylogenetically all the 20 isolated showed very minimal variations. Phylogenetic analysis using composition vectors showed that all 20 isolates are closely related, forming a close cluster with historical *V. cholerae* O1 El Tor strains from Haiti (2010–2012) and others. The same pattern was also observed using a core gene-based phylogeny. The close phylogenetic clustering and limited SNP diversity among isolates suggest the circulation of a genetically related outbreak lineage during the study period. The close phylogenetic relatedness of isolates collected from three districts within a short time frame suggests dissemination of a common outbreak-associated lineage during the 2024 Gujarat cholera outbreak. These findings support the utility of whole-genome sequencing for tracking outbreak dynamics and regional spread of *V. cholerae*. At the same time, the fact that both rise in the cases in Rajkot and Jamnagar and the wastewater samples tested positive, suggest that environmental contamination likely played a role in an outbreak. We lacked epidemiological linkage data between patients as well as we did not isolated *V. cholerae* from the wastewater to compare their genomes with those of the clinical isolates, such analysis could have provided a better understanding of the source and dynamics of the outbreak.

The continuously expanding pan-genome indicates an open pangenome, suggesting that new genes are consistently being introduced in the *V. cholerae*. This is characteristic of species with high genetic diversity, horizontal gene transfer, and ecological adaptability (Qin et al., 2025; Morgado et al., 2026; Uddin et al., 2026). And this could be one of the reasons for continuous cholera outbreaks worldwide. The presence of 2,618 core genes, including 732 soft-core genes, reflects a conserved set of essential genes that remain relatively constant in *V. cholerae*. This finding supports genomic studies that show *V. cholerae O*1 El Tor maintains a relatively stable core genome within the seventh pandemic (7PET) lineages, with genomic variability mainly driven by mobile elements such as ICEs, prophages, and integrons, rather than frequent acquisition of new genes (Chun et al., 2009; Mutreja et al., 2011). Our research highlights the “open but slowly expanding” characteristic of the *V. cholerae* pangenome. Although several isolates showed some unique genes, the absence of these genes in other isolates may be because of fragmented assemblies. Therefore, we recommend generating chromosome-level or single-contig level assemblies using long-reads sequencing to better resolve the true genomics architecture. Although new genes can come into play most likely through horizontal gene transfer, the epidemic O1 El Tor lineage tends to stay quite stable, with genetic variation limited to a small accessory pool. This stability is crucial from an epidemiological viewpoint, as it suggests that genomic surveillance can effectively track outbreak lineages using a consistent set of core markers, while also keeping an eye on the accessory genome for any sporadic gains of AMR genes or new phage elements.

Notably, resistance to ampicillin was observed in all 15 isolates for which antimicrobial susceptibility data was provided by the hospitals. However, for doxycycline, tetracycline, and gentamicin, the susceptibility we detected corresponds to the absence of related resistance genes in the genomes. The observed phenotype in this study for ampicillin (100%) is much higher compared to previous studies, where authors reported 48–65% isolated being resistant to it (Nateghizad et al., 2023; Al-Herwi et al., 2025; Chisompola et al., 2025). Furthermore, in opposed to this study, a meta-analysis from 2000 to 2020 also reported decreased resistance towards ampicillin, however, in non-O1/non-O139 *V. cholerae* (Wu et al., 2023). The observed resistance to cotrimoxazole (~85%) is aligning with 79% reported previously (Nateghizad et al., 2023). In case of susceptibility towards other antibiotics, the findings align with pervious meta-analysis from 2018–2024 in Ogawa Serotype (Al-Herwi et al., 2025). The antimicrobial determinants found in the current isolates are consistence with those previously reported in the seventh pandemic El Tor *V. cholerae* lineages that are circulating worldwide, especially in Asia and Africa. Recent genomics study from Bangladesh and other endemic areas have also noted the widespread presence of *dfrA1*, *sul2*, *floR*, and *catB9* in epidemic lineages (Mevada et al., 2023; Monir et al., 2023).

In the case of ciprofloxacin, isolates were phenotypically susceptible despite the presence of the *Escherichia coli parE* fluoroquinolone resistance determinant. Although all isolates carried the *parE* D476N mutation, the presence of this variant alone may not be sufficient to confer ciprofloxacin resistance, consistent with previous reports (Fuesslin et al., 2022). The findings for chloramphenicol are especially interesting. Despite detecting the *floR* and *catB9* genes, all 20 isolates remained phenotypically susceptible. Similar genotype–phenotype discordance has been reported previously (Fuesslin et al., 2022), where most isolates carried chloramphenicol resistance genes but remained susceptible in AST. This discrepancy may be due to regulatory suppression, non-functional gene, or insufficient efflux or acetyltransferase activity to overcome clinically relevant chloramphenicol concentrations. In addition, the position of the *catB9* cassette within the integron can critically influence expression; when located distally from the promoter, transcription may be insufficient to confer resistance, as demonstrated in *V. cholerae* by Rowe-Magnus et al. (Schwarz et al., 2004; Rowe-Magnus et al., 2002). The mixed resistance pattern for cotrimoxazole, with 3 out of 15 being susceptible and 12 showing resistance, indicates potential variability in the expression of *dfrA1* or other resistance mechanisms, necessitating further examination. Yet, the existence of *dfrA1* in integrons, primarily located in chromosomal areas, point out the influence of mobile genetic elements (MGEs) in the dissemination of resistance, aligning with worldwide trends noted in *V. cholerae* (Bhandari et al., 2021; De, 2021; Drebes Dörr et al., 2025). Such genotype–phenotype mismatches are well recognized in resistance surveillance, highlighting the importance of combining WGS with phenotypic AST for accurate clinical interpretation (Vanstokstraeten et al., 2023; Singh et al., 2025; Bhat et al., 2023). It is well established that gene cassettes located closer to the integron promoter are expressed more strongly, whereas those positioned farther away show progressively reduced expression (Ali et al., 2024; Gillings, 2014). In contrast to previous studies, the absence of CRISPR-Cas systems in isolates is a striking feature of the isolates (Box et al., 2016; McDonald et al., 2019).

Cholera toxin (CT) is a potent, multi-subunit enterotoxin secreted by *V. cholerae* that causes severe, dehydrating diarrhoea. CT is encoded by the *ctxAB* genes of the CTX prophage. Further, it is also interesting to note that not all 20 isolates have the *ctxA/ctxB* gene. A study from Assam, India also reported missing of *ctxA* gene 12% of isolates (Sharma et al., 2021). This is also in alignment with one of the recent studies where authors re-analysed more than 13,000 *V. cholerae* genomes and reported absence of cholera toxin in *V. paracholerae* (Morgado et al., 2026). However, all 20 isolates in this study possess several other virulence genes, including *hlyA* (Hemolysin A), *ace* (accessory cholera enterotoxin), *hapA* (Hemagglutinin/protease), *ompU* (Outer membrane protein U), and others. All these genes collectively enable *V. cholerae* to colonize the host, survive environmental and host-induced stresses, and cause disease. They also possess genes and virulent determinants related to adhesion and biofilm formation (MSHA pilus, *ompU*), regulate virulence expression and stress responses (*toxR*, *rpoS*, Lux operon), and promote tissue invasion and nutrient acquisition (*hapA*, *nanH*). Several genes encode toxins or toxic effectors that damage host cells and enhance pathogenicity, such as *ace*, *hlyA*, *makA*, *vasX* were also detected. Together, they coordinate host colonization, persistence, and host damage, ensuring the bacterium’s survival and transmission. Apart from these, a couple of isolates also carry the *zot* (Zonula Occludens Toxin) gene, encoding an enterotoxin that increases intestinal permeability (Uzzau et al., 1999), which leads to watery diarrhoea. It is also important to note that all isolates lack genes *chxA* (cholix toxin) and *stn* (Heat-Stable Enterotoxin), both of which are typically present in non-O1/non-O139 strains (Zhang et al., 2024).

The occurrence of integrative and conjugative elements (ICEs) in all isolates, each displaying 2 to 4 regions, highlights the impact of horizontal gene transfer on the resistome and virulome of *V. cholerae*. This again supports the open and flexible pan-genome of *V. cholerae*. It is noteworthy that integrons containing d*frA1* are regularly located in chromosomal region, except VP1, which corresponds with earlier research on the dissemination of integron-mediated resistance in *V. cholerae* (Marin et al., 2014; Sarkar et al., 2019). Additionally, the identification of transposases, integrases, and type IV secretion systems (T4SS) through VRprofile2 further supports the idea that antimicrobial resistance genes (ARGs) and virulence factors are quite mobile, raising concerns about the potential for further resistance to spread.

Importantly, wastewater surveillance findings were epidemiologically consistence with the clinical outbreak patten. A targeted PCR method for the most used marker for toxigenic *V. cholerae* (*ctxA*) was found in 20% (28/140) samples of wastewater in Rajkot and Jamnagar versus all negative from drinking water in Ahmedabad, highlighting the importance of wastewater surveillance for *V. cholerae*. The absence of *V. cholerae* in Ahmedabad drinking water suggests that the water treatment processes are working well, consistent with previous studies that have demonstrated that water quality plays a key role in controlling cholera (Luby et al., 2020; Chowdhury et al., 2025; Wen et al., 2025). Furthermore, our results align with recent suggestions that wastewater-based cholera surveillance can be valuable for improving case management during outbreaks and serve as a potential early warning system in routine surveillance (Panda et al., 2020). Since wastewater samples were collected during, and not prior to the outbreak, this study directly does not address the topic directly. Furthermore, although direct genomic comparison between clinical and environmental strains was not possible because *V. cholerae* was not isolated form the wastewater samples, the presence of it in wastewater suggest active environmental circulation of toxigenic *V. cholerae* during the outbreak. However, environmental surveillance, as opposed to clinical surveillance which relies on diagnosed and reported infections, detects pathogens shedding from all individuals, both those with and without symptoms, thus offering a more comprehensive view of pathogen circulation at population level (Tisza et al., 2023; Shukla N. et al., 2026). Continuous environmental monitoring enables the identification of changes in how abundance of pathogens varies across the time point. An abrupt rise in the abundance of the pathogen could be a sign of ongoing transmission in a particular area (Kumar et al., 2021b). Therefore, before widespread transmission take place, this early data enables public health authorities to carry out quick response and interventions.

There are some limitations to this study, particularly the lack of AST and serotyping data for all isolates, which limited comprehensive genotype–phenotype comparisons for all isolates we sequenced. The environmental analysis also had limitations because samples from Ahmedabad consisted only of drinking water, whereas those from Rajkot and Jamnagar were wastewater; therefore, direct comparisons between cities should be interpreted cautiously. Furthermore, the present findings do not establish direct transmission between environmental and clinical isolates, as environmental culture isolation and comparative genomic analysis were not performed. Therefore, while wastewater positivity and clinical case clustering showed harmony, definitive transmission linkage between environment and clinical strain could not be established. The effects of mutations such as *parE* D476N and EF-Tu R234F on resistance also require validation through functional studies or Sanger sequencing. The observed absence of certain genes in some isolates could be due to incomplete genome assemblies. Therefore, further whole sequencing using long-read sequencing, such as Oxford Nanopore, is recommended, which can generate chromosome-level assemblies. Future investigations should also explore the persistence of *V. cholerae* in the environment and the underlying factors contributing to the variations in resistance to chloramphenicol and cotrimoxazole. Nevertheless, because only a subset of isolates was selected for whole-genome sequencing, the full genomic diversity of the outbreak may not have been completely represented, and some degree of selection bias remains possible.

## 5 Conclusion

This study provides an in-depth analysis of *V. cholerae* O1 El Tor isolates from cholera outbreak in Gujarat in 2024 through genomic and phenotypic information. Although, the isolates are sensitive to most of antibiotics tested, except ampicillin and cotrimoxazole, the presence of ARGs and MGEs needs attention, especially for the transfer of antimicrobial-resistant and virulence genes. The findings also highlight the critical need for effective wastewater management, the proliferation of multidrug-resistant and the importance of genomic surveillance in managing outbreaks. These results contribute to a broader understanding of cholera epidemiology globally and inform targeted public health initiatives in regions where cholera is prevalent.

## Data Availability

The data including raw sequencing FASTQ and genome assemblies are deposited in NCBI under the BioProject PRJEB96384 and individual genome accessions are provided in Table 1.

## References

[ref9001] Ali N. Ali I. Din A. U. Akhtar K. He B. Wen R. (2024). Integrons in the Age of Antibiotic Resistance: Evolution, Mechanisms, and Environmental Implications: A Review. Microorganisms. 12. doi: 10.3390/microorganisms12122579 PMC11676243 39770781

[ref1] Albert M. J. (1996). Epidemiology & molecular biology of *Vibrio cholerae* O139 Bengal. Indian J. Med. Res. 104, 14–27. 8783504

[ref2] Al-Herwi E. Al-Kainai H. Alshoaibi M. Thabet H. Farhan M. A. Morshed E. . (2025). Outbreak of multi-drug resistant *Vibrio cholerae* Ogawa serotype in Ibb, Yemen (2018–2024): the OVCO-IY study. Int. J. Infect. Dis. 161:108134. doi: 10.1016/j.ijid.2025.108134, 41110589

[ref3] Alikhan N.-F. Petty N. K. Ben Zakour N. L. Beatson S. A. (2011). BLAST ring image generator (BRIG): simple prokaryote genome comparisons. BMC Genomics 12:402. doi: 10.1186/1471-2164-12-402, 21824423 PMC3163573

[ref4] Argimón S. David S. Underwood A. Abrudan M. Wheeler N. E. Kekre M. . (2021). Rapid genomic characterization and global surveillance of *Klebsiella* using Pathogenwatch. Clin. Infect. Dis. 73, S325–S335. doi: 10.1093/cid/ciab784, 34850838 PMC8634497

[ref5] Ayyappan M. V. Kishore P. Panda S. K. Kumar A. Uchoi D. Nadella R. K. . (2024). Emergence of multidrug resistant, ctx negative seventh pandemic *Vibrio cholerae* O1 El Tor sequence type (ST) 69 in coastal water of Kerala, India. Sci. Rep. 14:2031. doi: 10.1038/s41598-023-50536-z, 38263228 PMC10805778

[ref9002] Bhat B. A. Mir R. A. Qadri H. Dhiman R. Almilaibary A. Alkhanani M. (2023). Integrons in the development of antimicrobial resistance: critical review and perspectives. Front. Microbiol. 14:2023. doi: 10.3389/fmicb.2023.1231938 PMC10500605 37720149

[ref6] Bhandari M. Jennison A. V. Rathnayake I. U. Huygens F. (2021). Evolution, distribution and genetics of atypical *Vibrio cholerae*—a review. Infect. Genet. Evol. 89:104726. doi: 10.1016/j.meegid.2021.104726 33482361

[ref7] Box A. M. McGuffie M. J. O'Hara B. J. Seed K. D. (2016). Functional analysis of bacteriophage immunity through a type I-E CRISPR-Cas system in *Vibrio cholerae* and its application in bacteriophage genome engineering. J. Bacteriol. 198, 578–590. doi: 10.1128/jb.00747-15, 26598368 PMC4719448

[ref8] Bu C. Zhang H. Zhang F. Liang W. Gao H. Zhao J. . (2025). PGAP2: a comprehensive toolkit for prokaryotic pan-genome analysis based on fine-grained feature networks. Nat. Commun. 16:9865. doi: 10.1038/s41467-025-64846-5, 41213900 PMC12603064

[ref9] Cantu V. A. Sadural J. Edwards R. (2019). PRINSEQ++, a multi-threaded tool for fast and efficient quality control and preprocessing of sequencing datasets. PeerJ. doi: 10.7287/peerj.preprints.27553v1

[ref10] CD Genomics. (2025). Pan-genome: definition, classification, and why it matters. Available online at: https://www.cd-genomics.com/pop-genomics/resources/pan-genome-definition-classification.html (accessed May 27, 2026).

[ref11] Chaguza C. Chibwe I. Chaima D. Musicha P. Ndeketa L. Kasambara W. . (2024). Genomic insights into the 2022–2023*Vibrio cholerae* outbreak in Malawi. Nat. Commun. 15:6291. doi: 10.1038/s41467-024-50484-w, 39060226 PMC11282309

[ref12] Chigwechokha P. Nyirenda R. L. Dalitsani D. Namaumbo R. L. Kazembe Y. Smith T. . (2024). Vibrio cholerae and *Salmonella Typhi* culture-based wastewater or non-sewered sanitation surveillance in a resource-limited region. J. Expo. Sci. Environ. Epidemiol. 34, 432–439. doi: 10.1038/s41370-023-00632-z, 38177335

[ref13] Chisompola D. Nzobokela J. Moono R. Chinyante E. Chipipa A. Chapuswike N. . (2025). Emerging antibiotic resistance in *Vibrio cholerae*: a study of cholera prevalence and resistance patterns in Zambia’s Copperbelt Province. BMC Infect. Dis. 25:879. doi: 10.1186/s12879-025-11259-w, 40597869 PMC12220150

[ref14] Chowdhury M. R. Islam A. Yurina V. Shimosato T. (2025). Water pollution, cholera, and the role of probiotics: a comprehensive review in relation to public health in Bangladesh. Front. Microbiol. 15:1523397. doi: 10.3389/fmicb.2024.1523397 39877756 PMC11772269

[ref15] Chun J. Grim C. J. Hasan N. A. Lee J. H. Choi S. Y. Haley B. J. . (2009). Comparative genomics reveals mechanism for short-term and long-term clonal transitions in pandemic *Vibrio cholerae*. Proc. Natl. Acad. Sci. 106, 15442–15447. doi: 10.1073/pnas.0907787106, 19720995 PMC2741270

[ref16] Couvin D. Bernheim A. Toffano-Nioche C. Touchon M. Michalik J. Néron B. . (2018). CRISPRCasFinder, an update of CRISRFinder, includes a portable version, enhanced performance and integrates search for Cas proteins. Nucleic Acids Res. 46, W246–W251. doi: 10.1093/nar/gky425, 29790974 PMC6030898

[ref17] De R. (2021). Mobile genetic elements of *Vibrio cholerae* and the evolution of its antimicrobial resistance. Front. Trop. Dis. 2:691604. doi: 10.3389/fitd.2021.691604

[ref18] Domman D. Quilici M.-L. Dorman M. J. Njamkepo E. Mutreja A. Mather A. E. . (2017). Integrated view of *Vibrio cholerae* in the Americas. Science 358, 789–793. doi: 10.1126/science.aao2136 29123068

[ref19] Donatella L. Eduardo G. Saverio C. (2016). Cholera. Microbiol. Spectrum 4, 1–6. doi: 10.1128/microbiolspec.poh-0012-2015, 27726771

[ref20] Drebes Dörr N. C. Lemopoulos A. Blokesch M. (2025). Exploring Mobile genetic elements in *Vibrio cholerae*. Genome Biol. Evol. 17:evaf079. doi: 10.1093/gbe/evaf079, 40302206 PMC12082036

[ref21] Faruque S. M. Sack D. A. Sack R. B. Colwell R. R. Takeda Y. Nair G. B. (2003). Emergence and evolution of *Vibrio cholerae* O139. Proc. Nat. Acad. Sci. 100, 1304–1309. doi: 10.1073/pnas.0337468100, 12538850 PMC298768

[ref22] Finkelstein R. A. (1996). “Cholera, *Vibrio cholerae* O1 and O139, and other pathogenic Vibrios.,” in Medical Microbiology, ed. Baron S. (Galveston: University of Texas Medical Branch at Galveston). Available online at: https://www.ncbi.nlm.nih.gov/books/NBK8407/ (accessed February 16, 2026). 21413330

[ref23] Fuesslin V. Krautwurst S. Srivastava A. Winter D. Liedigk B. Thye T. . (2022). Prediction of antibiotic susceptibility profiles of *Vibrio cholerae* isolates from whole genome Illumina and Nanopore sequencing data: CholerAegon. Front. Microbiol. 13:909692. doi: 10.3389/fmicb.2022.909692, 35814690 PMC9257098

[ref9003] Gillings M. R. (2014). Integrons: Past, Present, and Future. Microbiology and Molecular Biology Reviews 78, 257–277. doi: 10.1128/mmbr.00056-13 24847022 PMC4054258

[ref24] Hasan N. A. Choi S. Y. Eppinger M. Clark P. W. Chen A. Alam M. . (2012). Genomic diversity of 2010 Haitian cholera outbreak strains. Proc. Nat. Acad. Sci. 109, E2010–E2017. doi: 10.1073/pnas.1207359109, 22711841 PMC3406840

[ref25] Hoshino K. Yamasaki S. Mukhopadhyay A. K. Chakraborty S. Basu A. Bhattacharya S. K. . (1998). Development and evaluation of a multiplex PCR assay for rapid detection of toxigenic *Vibrio cholerae* O1 and O139. FEMS Immunol. Med. Microbiol. 20, 201–207. doi: 10.1111/j.1574-695X.1998.tb01128.x, 9566491

[ref26] Hossain M. J. Rasin J. H. Altaib H. Shyama I. J. Shawon R. A. R. Badr Y. . (2025). Prevalence and antibiotic resistance patterns of *Vibrio cholerae* isolated from diverse food sources in Khulna, Bangladesh. Food Humanity 5:100653. doi: 10.1016/j.foohum.2025.100653

[ref27] Irenge L. M. Ambroise J. Bearzatto B. Durant J.-F. Bonjean M. Wimba L. K. . (2024). Genomic evolution and rearrangement of CTX-Φ prophage elements in *Vibrio cholerae* during the 2018–2024 cholera outbreaks in eastern Democratic Republic of the Congo. Emerg. Microbes Infect. 13:2399950. doi: 10.1080/22221751.2024.2399950, 39259213 PMC11395875

[ref28] Irenge L. M. Ambroise J. Mitangala P. N. Bearzatto B. Kabangwa R. K. S. Durant J.-F. . (2020). Genomic analysis of pathogenic isolates of *Vibrio cholerae* from eastern Democratic Republic of the Congo (2014-2017). PLoS Negl. Trop. Dis. 14:e0007642. doi: 10.1371/journal.pntd.0007642, 32310947 PMC7192507

[ref29] Jaeyres J. Leaslie J. J. Natasha A. L. Yebon K. D. Montini M. M. C. Mohammad J. . (2026). Atypical El Tor *Vibrio cholerae* from the second major global seventh-pandemic cholera wave is endemic in Sabah, Malaysia. Microbiol. Spectrum 14:e02191-25. doi: 10.1128/spectrum.02191-25, 41660980 PMC12955491

[ref30] Jolley K. A. Bray J. E. Maiden M. C. J. (2018). Open-access bacterial population genomics: BIGSdb software, the PubMLST.org website and their applications. Wellcome Open Res. 3:124. doi: 10.12688/wellcomeopenres.14826.1, 30345391 PMC6192448

[ref31] Kachwamba Y. Mohammed A. A. Lukupulo H. Urio L. Majigo M. Mosha F. . (2017). Genetic characterization of *Vibrio cholerae* O1 isolates from outbreaks between 2011 and 2015 in Tanzania. BMC Infect. Dis. 17:157. doi: 10.1186/s12879-017-2252-9, 28219321 PMC5319185

[ref32] Kumar M. Joshi M. Patel A. K. Joshi C. G. (2021a). Unravelling the early warning capability of wastewater surveillance for COVID-19: A temporal study on SARS-CoV-2 RNA detection and need for the escalation. Environ. Res. 196:110946. doi: 10.1016/j.envres.2021.110946, 33662347 PMC7921726

[ref33] Kumar M. Joshi M. Shah A. V. Srivastava V. Dave S. (2021b). Wastewater surveillance-based city zonation for effective COVID-19 pandemic preparedness powered by early warning: a perspectives of temporal variations in SARS-CoV-2-RNA in Ahmedabad, India. Sci. Total Environ. 792:148367. doi: 10.1016/j.scitotenv.2021.148367, 34465041 PMC8186940

[ref34] Lassalle F. Al-Shalali S. Al-Hakimi M. Njamkepo E. Bashir I. M. Dorman M. J. . (2023). Genomic epidemiology reveals multidrug resistant plasmid spread between *Vibrio cholerae* lineages in Yemen. Nat. Microbiol. 8, 1787–1798. doi: 10.1038/s41564-023-01472-1, 37770747 PMC10539172

[ref35] Legros D. (2018). Global cholera epidemiology: opportunities to reduce the burden of cholera by 2030. J. Infect. Dis. 218, S137–S140. doi: 10.1093/infdis/jiy486, 30184102 PMC6207143

[ref36] Letunic I. Bork P. (2024). Interactive tree of life (iTOL) v6: recent updates to the phylogenetic tree display and annotation tool. Nucleic Acids Res. 52, W78–W82. doi: 10.1093/nar/gkae268, 38613393 PMC11223838

[ref37] Li W. Sun Y. Ma T. Lu W. Sa N. Gong L. . (2025). Genomic characteristics and antibiotic resistance evolution of *Vibrio cholerae* O139-Anhui Province, China, 2013–2024. China CDC Wkly. 7, 1057–1063. doi: 10.46234/ccdcw2025.179, 40837136 PMC12361915

[ref38] Liam C. Michael P. Sandeep K. Ruiting L. (2021). Multilevel genome typing describes short- and long-term *Vibrio cholerae* molecular epidemiology. mSystems 6:10.1128/msystems.00134-21. doi: 10.1128/msystems.00134-21, 34427512 PMC8407458

[ref39] Liang Q. Liu C. Xu R. Song M. Zhou Z. Li H. . (2021). fIDBAC: a platform for fast bacterial genome identification and typing. Front. Microbiol. 12:723577. doi: 10.3389/fmicb.2021.723577, 34733246 PMC8558511

[ref40] Lippi D. Gotuzzo E. (2014). The greatest steps towards the discovery of *Vibrio cholerae*. Clin. Microbiol. Infect. 20, 191–195. doi: 10.1111/1469-0691.12390, 24191858

[ref41] Liu C. Wang Y. Azizian K. Omidi N. Hassan Kaviar V. Kouhsari E. . (2022). Antimicrobial resistance in *Vibrio cholerae* O1/O139 clinical isolates: a systematic review and meta-analysis. Expert Rev. Anti-Infect. Ther. 20, 1217–1231. doi: 10.1080/14787210.2022.2098114, 35790112

[ref42] Luby S. P. Davis J. Brown R. R. Gorelick S. M. Wong T. H. F. (2020). Broad approaches to cholera control in Asia: water, sanitation and handwashing. Vaccine 38, A110–A117. doi: 10.1016/j.vaccine.2019.07.084, 31383486

[ref43] Luo Y. Payne M. Kaur S. Octavia S. Lan R. (2024). Genomic evidence of two-staged transmission of the early seventh cholera pandemic. Nat. Commun. 15:8504. doi: 10.1038/s41467-024-52800-w, 39353924 PMC11445481

[ref44] Mageto L. M. Aboge G. O. Mekuria Z. H. Gathura P. Juma J. Mugo M. . (2025). Genomic characterization of *Vibrio cholerae* isolated from clinical and environmental sources during the 2022–2023 cholera outbreak in Kenya. Front. Microbiol. 16:1603736. doi: 10.3389/fmicb.2025.1603736, 40693147 PMC12277283

[ref45] Manna T. Chandra Guchhait K. Jana D. Dey S. Karmakar M. Hazra S. . (2024). Wastewater-based surveillance of *Vibrio cholerae*: molecular insights on biofilm regulatory diguanylate cyclases, virulence factors and antibiotic resistance patterns. Microb. Pathog. 196:106995. doi: 10.1016/j.micpath.2024.106995, 39368563

[ref46] Manni M. Berkeley M. R. Seppey M. Zdobnov E. M. (2021). BUSCO: assessing genomic data quality and beyond. Curr. Protoc. 1:e323. doi: 10.1002/cpz1.323, 34936221

[ref47] Marin M. A. Fonseca E. L. Andrade B. N. Cabral A. C. Vicente A. C. P. (2014). Worldwide occurrence of integrative conjugative element encoding multidrug resistance determinants in epidemic *Vibrio cholerae* O1. PLoS One 9:e108728. doi: 10.1371/journal.pone.0108728, 25265418 PMC4181655

[ref48] Matteo S. Daniela C. Adrien R. Marco F. Elisa T. Renato F. . (2014). Acquisition and evolution of SXT-R391 integrative conjugative elements in the seventh-pandemic *Vibrio cholerae* lineage. MBio 5:e01356-14. doi: 10.1128/mbio.01356-14, 25139901 PMC4147863

[ref49] McArthur A. G. Waglechner N. Nizam F. Yan A. Azad M. A. Baylay A. J. . (2013). The comprehensive antibiotic resistance database. Antimicrob. Agents Chemother. 57, 3348–3357. doi: 10.1128/aac.00419-13, 23650175 PMC3697360

[ref50] McDonald N. D. Regmi A. Morreale D. P. Borowski J. D. Boyd E. F. (2019). CRISPR-Cas systems are present predominantly on mobile genetic elements in Vibrio species. BMC Genomics 20:105. doi: 10.1186/s12864-019-5439-1, 30717668 PMC6360697

[ref51] Meier-Kolthoff J. P. Göker M. (2019). TYGS is an automated high-throughput platform for state-of-the-art genome-based taxonomy. Nat. Commun. 10:2182. doi: 10.1038/s41467-019-10210-3, 31097708 PMC6522516

[ref52] Mevada V. Patel R. Dudhagara P. Chaudhari R. Vohra M. Khan V. . (2023). Whole genome sequencing and Pan-genomic analysis of multidrug-resistant *Vibrio cholerae* VC01 isolated from a clinical sample. Microorganisms 11:2030. doi: 10.3390/microorganisms11082030, 37630590 PMC10457874

[ref53] Mohiuddin K. M. Rayhan I. M. Zinat F. Faiz H. C. (2023). Complete genome sequence of the pandrug-resistant *Vibrio cholerae* strain KBR06 isolated from a cholera patient in Bangladesh. Microbiol. Resour. Announc. 12:e00577-23. doi: 10.1128/MRA.00577-23, 37966233 PMC10720518

[ref54] Monir M. M. Islam M. T. Mazumder R. Mondal D. Nahar K. S. Sultana M. . (2023). Genomic attributes of *Vibrio cholerae* O1 responsible for 2022 massive cholera outbreak in Bangladesh. Nat. Commun. 14:1154. doi: 10.1038/s41467-023-36687-7, 36859426 PMC9977884

[ref55] Montero D. A. Vidal R. M. Velasco J. George S. Lucero Y. Gómez L. A. . (2023). *Vibrio cholerae*, classification, pathogenesis, immune response, and trends in vaccine development. Front. Med. 10:1155751. doi: 10.3389/fmed.2023.1155751, 37215733 PMC10196187

[ref56] Morgado S. M. da Fonseca E. L. Vicente A. C. P. (2026). Tracking a misclassified pathogen: genomic and epidemiological features of *Vibrio paracholerae*. Microb. Genom. 12:001605. doi: 10.1099/mgen.0.001605, 41525209 PMC12795556

[ref57] Morris J. G. Jr. (1995). *Vibrio cholerae* O139 Bengal: emergence of a new epidemic strain of cholera. Infect. Agents Dis. 4, 41–46. 7728355

[ref58] Mutreja A. Kim D. W. Thomson N. R. Connor T. R. Lee J. H. Kariuki S. . (2011). Evidence for several waves of global transmission in the seventh cholera pandemic. Nature 477, 462–465. doi: 10.1038/nature10392, 21866102 PMC3736323

[ref59] Muzembo B. A. Kitahara K. Debnath A. Ohno A. Okamoto K. Miyoshi S.-I. (2022). Cholera outbreaks in India, 2011–2020: a systematic review. Int. J. Environ. Res. Public Health 19:5738. doi: 10.3390/ijerph19095738, 35565133 PMC9099871

[ref60] Nair S. Barker C. R. Patel V. Poh C.-Y. Greig D. R. Olonade I. . (2025). Highly drug-resistant *Vibrio cholerae* harbouring blaPER-7 isolated from travellers returning to England. J. Antimicrob. Chemother. 80, 2428–2432. doi: 10.1093/jac/dkaf232, 40671289 PMC12404714

[ref61] Nateghizad H. Sajadi R. Shivaee A. Shirazi O. Sharifian M. Tadi D. A. . (2023). Resistance of *Vibrio cholera* to antibiotics that inhibit cell wall synthesis: a systematic review and meta-analysis. Front. Pharmacol. 14:1027277. doi: 10.3389/fphar.2023.1027277, 37021056 PMC10069679

[ref62] Panda S. Chatterjee P. Deb A. Kanungo S. Dutta S. (2020). Preventing cholera in India: synthesizing evidences through a systematic review for policy discussion on the use of oral cholera vaccine. Vaccine 38, A148–A156. doi: 10.1016/j.vaccine.2019.07.029, 31405636

[ref63] Parks D. H. Imelfort M. Skennerton C. T. Hugenholtz P. Tyson G. W. (2015). CheckM: assessing the quality of microbial genomes recovered from isolates, single cells, and metagenomes. Genome Res. 25, 1043–1055. doi: 10.1101/gr.186072.114, 25977477 PMC4484387

[ref64] Parvin I. Shahid A. S. M. S. B. Das S. Shahrin L. Ackhter M. M. Alam T. . (2021). *Vibrio cholerae* O139 persists in Dhaka, Bangladesh since 1993. PLoS Negl. Trop. Dis. 15:e0009721. doi: 10.1371/journal.pntd.0009721, 34473699 PMC8443037

[ref65] Pourmohsenin B. Wiese A. Ziemert N. (2025). AutoMLST2: a web server for phylogeny and microbial taxonomy. Nucleic Acids Res. 53, W45–W50. doi: 10.1093/nar/gkaf397, 40357641 PMC12230714

[ref66] Qi J. Wang B. Hao B.-I. (2004). Whole proteome prokaryote phylogeny without sequence alignment: a K-string composition approach. J. Mol. Evol. 58, 1–11. doi: 10.1007/s00239-003-2493-7 14743310

[ref67] Qin C. Lypaczewski P. Sayeed M. A. Cuénod A. Brinkley L. Creasy-Marrazzo A. . (2025). *Vibrio cholerae* lineage and pangenome diversity vary geographically across Bangladesh over 1 year. Microb. Genom. 11:001437. doi: 10.1099/mgen.0.001437, 40709915 PMC12452190

[ref68] Ramamurthy T. Mutreja A. Weill F.-X. Das B. Ghosh A. Nair G. B. (2019). Revisiting the global epidemiology of cholera in conjunction with the genomics of *Vibrio cholerae*. Front. Public Health 7:237. doi: 10.3389/fpubh.2019.00203, 31396501 PMC6664003

[ref69] Ramamurthy T. Pragasam A. K. Taylor-Brown A. Will R. C. Vasudevan K. Das B. . (2022). *Vibrio cholerae* O139 genomes provide a clue to why it may have failed to usher in the eighth cholera pandemic. Nat. Commun. 13:3864. doi: 10.1038/s41467-022-31391-4, 35790755 PMC9256687

[ref9005] Rowe-Magnus D. A. Guerout A.-M. Mazel D. (2002). Bacterial resistance evolution by recruitment of super-integron gene cassettes. Mol. Microbiol. 43, 1657–1669. doi: 10.1046/j.1365-2958.2002.02861.x 11952913

[ref70] Sabir D. K. Hama Z. T. Salih K. J. Khidhir K. G. (2023). A molecular and epidemiological study of cholera outbreak in Sulaymaniyah Province, Iraq, in 2022. Pol. J. Microbiol. 72, 39–46. doi: 10.33073/pjm-2023-008, 36929893 PMC10280330

[ref71] Samal D. Turuk J. Nayak S. R. Pany S. Pal B. B. Pati S. (2025). Genomic insights into the dynamic antibiotic resistance landscape of *Vibrio cholerae* during the cholera outbreak 2022 in Odisha, India. Sci. Rep. 15:1503. doi: 10.1038/s41598-024-81596-4, 39789042 PMC11718308

[ref72] Sarkar A. Morita D. Ghosh A. Chowdhury G. Mukhopadhyay A. K. Okamoto K. . (2019). Altered integrative and conjugative elements (ICEs) in recent *Vibrio cholerae* O1 isolated from cholera cases, Kolkata, India. Front. Microbiol. 10:2072. doi: 10.3389/fmicb.2019.02072, 31555253 PMC6743048

[ref9006] Schwarz S. Kehrenberg C. Doublet B. Cloeckaert A. (2004). Molecular basis of bacterial resistance to chloramphenicol and florfenicol. FEMS Microbiol. Rev. 28, 519–542. doi: 10.1016/j.femsre.2004.04.001 15539072

[ref73] Sharma A. Sarmah Dutta B. Rabha D. Samsun Rasul E. Kumar Hazarika N. (2021). Molecular characterization of *Vibrio cholerae* O1 strains circulating in Assam: a north eastern state of India. Iran. J. Microbiol. 13, 583–591. doi: 10.18502/ijm.v13i5.7420, 34900155 PMC8629826

[ref74] Shukla N. Thakor J. Chavda P. Purohit H. Patel H. Thakar S. . (2026). Longitudinal wastewater virome surveillance unveils untapped circulating viruses in the community. NPJ Emerg. Contam. 2:15. doi: 10.1038/s44454-026-00035-3

[ref75] Shukla M. Wattam A. R. Aleman A. Bhattacharya R. Bowers N. Brettin T. . (2026). BV-BRC: a unified bacterial and viral bioinformatics resource with expanded functionality and AI integration. Nucleic Acids Res. 54, D715–D723. doi: 10.1093/nar/gkaf1254, 41251138 PMC12807693

[ref76] Simão F. A. Waterhouse R. M. Ioannidis P. Kriventseva E. V. Zdobnov E. M. (2015). BUSCO: assessing genome assembly and annotation completeness with single-copy orthologs. Bioinformatics 31, 3210–3212. doi: 10.1093/bioinformatics/btv351, 26059717

[ref9007] Singh H. Pandya S. Jasani S. Patel M. Kaur T. Rustagi S. (2025). Integrons: the hidden architects of bacterial adaptation, evolution, and the challenges of antimicrobial resistance. Antonie Van Leeuwenhoek 118, 90. doi: 10.1007/s10482-025-02103-x 40506592

[ref77] Tisza M. Javornik Cregeen S. Avadhanula V. Zhang P. Ayvaz T. Feliz K. . (2023). Wastewater sequencing reveals community and variant dynamics of the collective human virome. Nat. Commun. 14:6878. doi: 10.1038/s41467-023-42064-1, 37898601 PMC10613200

[ref78] Uddin M. S. Chamonara K. Masum M. H. U. Siddiqua A. Hossain I. Sultana S. . (2026). Genomic and phylogenomic exploration of *Vibrio cholerae* strain SU129B isolated from *Penaeus vannamei*: resistance, virulence, and evolutionary dynamics in bangladeshi aquaculture. PLoS One 21:e0344787. doi: 10.1371/journal.pone.0344787, 41805947 PMC12974878

[ref79] Uzzau S. Cappuccinelli P. Fasano A. (1999). Expression of *Vibrio cholerae* zonula occludens toxin and analysis of its subcellular localization. Microb. Pathog. 27, 377–385. doi: 10.1006/mpat.1999.0312 10588910

[ref9004] Vanstokstraeten R. Piérard D. Crombé F. De-Geyter D. Wybo I. Muyldermans A. (2023). Genotypic resistance determined by whole genome sequencing versus phenotypic resistance in 234 Escherichia coli isolates. Sci. Rep. 13, 449. doi: 10.1038/s41598-023-27723-z 36624272 PMC9829913

[ref80] Wang M. Goh Y.-X. Tai C. Wang H. Deng Z. Ou H.-Y. (2022). VRprofile2: detection of antibiotic resistance-associated mobilome in bacterial pathogens. Nucleic Acids Res. 50, W768–W773. doi: 10.1093/nar/gkac321, 35524563 PMC9252795

[ref81] Wang M. Liu G. Liu M. Tai C. Deng Z. Song J. . (2024). ICEberg 3.0: functional categorization and analysis of the integrative and conjugative elements in bacteria. Nucleic Acids Res. 52, D732–D737. doi: 10.1093/nar/gkad935, 37870467 PMC10767825

[ref82] Wen W. Zhao W. Zheng D. Chen J. Kan B. Zhou H. . (2025). Water and sanitation access shapes cholera burden in low- and middle-income countries. Glob. Transit. 7, 333–341. doi: 10.1016/j.glt.2025.06.001

[ref83] Wick R. R. Judd L. M. Gorrie C. L. Holt K. E. (2017). Unicycler: resolving bacterial genome assemblies from short and long sequencing reads. PLoS Comput. Biol. 13:e1005595. doi: 10.1371/journal.pcbi.1005595, 28594827 PMC5481147

[ref84] Wu Q. Vaziri A. Z. Omidi N. Hassan Kaviar V. Maleki A. Khadivar P. . (2023). Antimicrobial resistance among clinical *Vibrio cholerae* non-O1/non-O139 isolates: systematic review and meta-analysis. Pathog. Glob. Health 117, 235–244. doi: 10.1080/20477724.2022.2114620, 35983997 PMC10081078

[ref85] Yuan X. Li Y. Vaziri A. Z. Kaviar V. H. Jin Y. Jin Y. . (2022). Global status of antimicrobial resistance among environmental isolates of *Vibrio cholerae* O1/O139: a systematic review and meta-analysis. Antimicrob. Resist. Infect. Control 11:62. doi: 10.1186/s13756-022-01100-3, 35468830 PMC9036709

[ref86] Yun L. Michael P. Sandeep K. Sophie O. Jianmin J. Ruiting L. (2023). Emergence and genomic insights of non-pandemic O1 *Vibrio cholerae* in Zhejiang, China. Microbiol. Spectrum 11:e02615-23. doi: 10.1128/spectrum.02615-23, 37819129 PMC10871787

[ref87] Zhang Q. Alter T. Strauch E. Eichhorn I. Borowiak M. Deneke C. . (2024). German coasts harbor non-O1/non-O139 *Vibrio cholerae* with clinical virulence gene profiles. Infect. Genet. Evol. 120:105587. doi: 10.1016/j.meegid.2024.105587, 38518953

[ref88] Zhou S. Liu B. Zheng D. Chen L. Yang J. (2025). VFDB 2025: an integrated resource for exploring anti-virulence compounds. Nucleic Acids Res. 53, D871–D877. doi: 10.1093/nar/gkae968, 39470738 PMC11701737

[ref89] Zuo G. (2021). CVTree: a parallel alignment-free phylogeny and taxonomy tool based on composition vectors of genomes. Genomics Proteomics Bioinformatics 19, 662–667. doi: 10.1016/j.gpb.2021.03.006, 34119695 PMC9040009

