# Supplementary material for: Genome-wide investigation of outbreak-associated Vibrio cholerae in Gujarat, India identifies antimicrobial resistance genes, virulence determinants, and mobile genetic elements
Source: Front Microbiol. 2026 Jul 2;17:1851551. doi: 10.3389/fmicb.2026.1851551 (PMC13373057; doi:10.3389/fmicb.2026.1851551)
Supplement: Supplementary file 1 [file Data_Sheet_1.zip › Supplementary Figures.docx]

**Genome-wide investigation of outbreak-associated *Vibrio cholerae* in Gujarat, India identifies antimicrobial resistance genes, virulence determinants, and mobile genetic elements**

**Minal Bhure^1^, Nitin Shukla^1^, Harshal Purohit^1^, Nimesh Patel^1^, Priyank Chavda^1^, Madhulika Mistry^2^, Hitesh Shingala^3^, Bhavin Solanki^4^, Chirag Shah^5^, Madhvi Joshi^1,7^ and Chaitanya Joshi^1,6^, Snehal Bagatharia^*1^, Ramesh Pandit^*1^**

^1^Gujarat Biotechnology Research Centre, Department of Science and Technology, Government of Gujarat, 6^th^ Floor MS Building, Sector-11, Gandhinagar, Gujarat-382 010, India.

^2^Pandit Deendayal Upadhyay Medical College, Civil Hospital campus, Rajkot, Gujarat-360 001, India.

^3^M.P. Shah Govt. Medical College, Bedi Road, Pandit Nehru Marg, Indradeep Society, Jamnagar, Gujarat-361 008, India.

^4^Ahmedabad Municipal Corporation (AMC), Health Department, Sardar Patel Bhavan, Danapith, Ahmedabad, Gujarat-380 001, India.

^5^Ahmedabad Municipal Corporation (AMC), Health Department, VBDC Branch, 1st Floor, Aarogya Bhavan, Ahmedabad, Gujarat-380 022, India.

^6^Department of Veterinary Biotechnology, College of Veterinary Science and Animal Husbandry, Kamdhenu University, Anand, Gujarat-388 001, India.

^7^Hester Biosciences Limited, Meda-Adraj, Kadi, Mehsana, Gujarat-384 441, India.

Correspondence: **Ramesh Pandit:** [ramesh.scib@gbrc.res.in](mailto:ramesh.scib@gbrc.res.in), **Snehal Bagtharia:** [director@gbrc.res.in](mailto:director@gbrc.res.in)

# These authors have equal contributions †


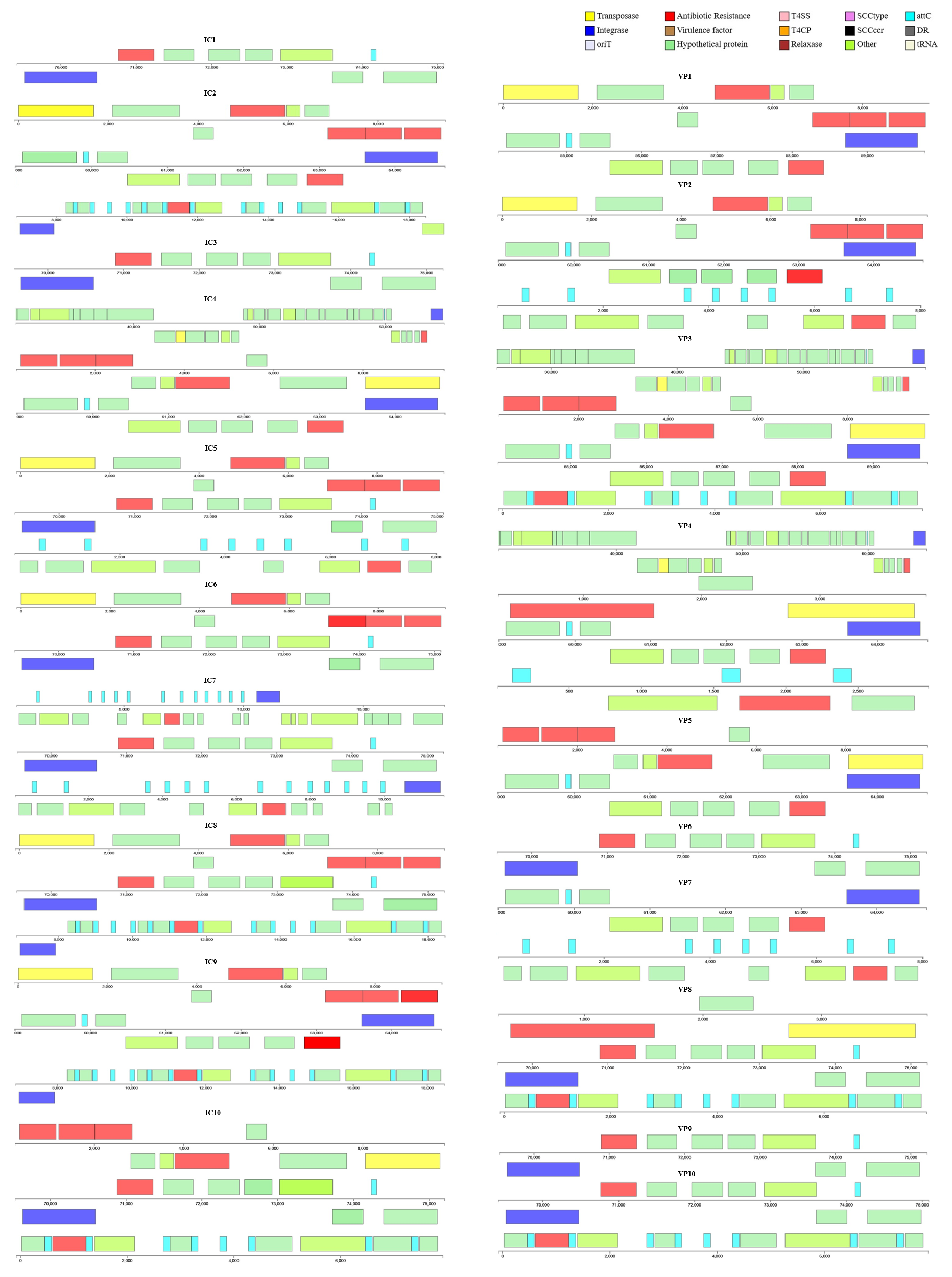


**Supplementary figure S1:** Depicting presence of mobile genetic elements in all 20 *V. cholerae* isolates.


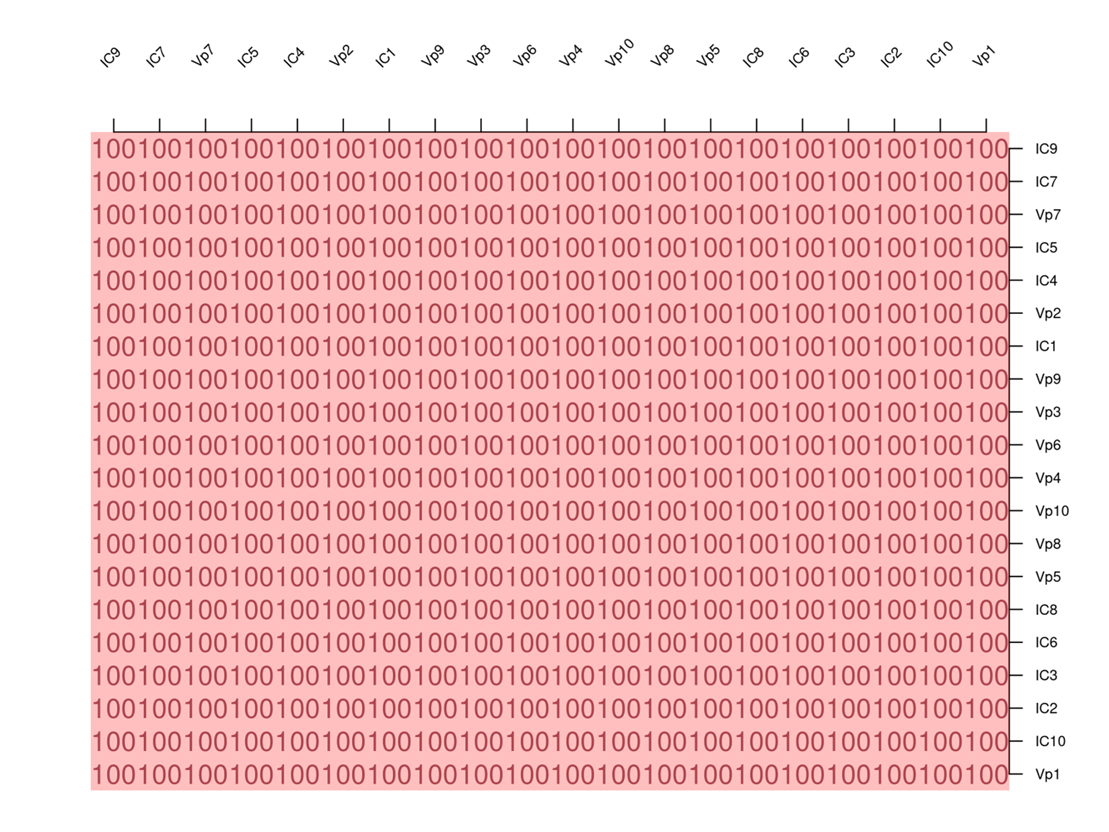


**Supplementary figure S2:** Depicting 100% average nucleotide identity (ANI-AAI matrix). ANI was calculated using ANI/AAI-Matrix Genome-based distance matrix calculator available at <http://enve-omics.ce.gatech.edu/g-matrix/>.


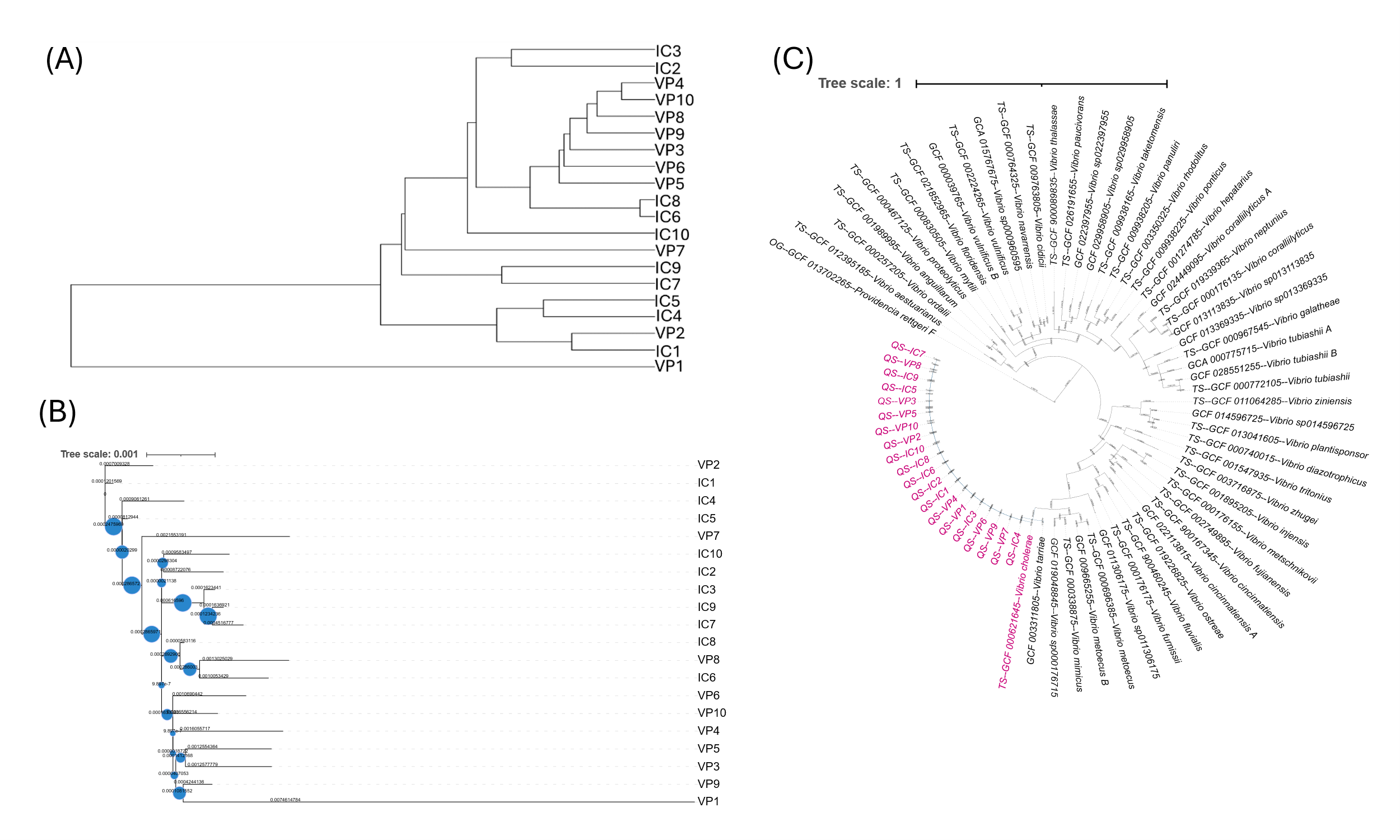


**Supplementary figure S3:** Phylogeny using different methods. (A) ANI-based phylogeny, (B) SNP-based phylogeny, and (C) multi-locus sequence-based phylogeny. ANI-based phylogeny was analysed using ANI/AAI-Matrix Genome-based distance matrix calculator. SNP-based phylogeny was analysed using fiDBAC SNP calling and workflow integrating maximum likelihood tree. autoMLST2.0 was also used build phylogeny based on MLST genes. In figure (B), SNP-based phylogeny, the size of blue colour dots are proportional to bootstrap values from 10-100.


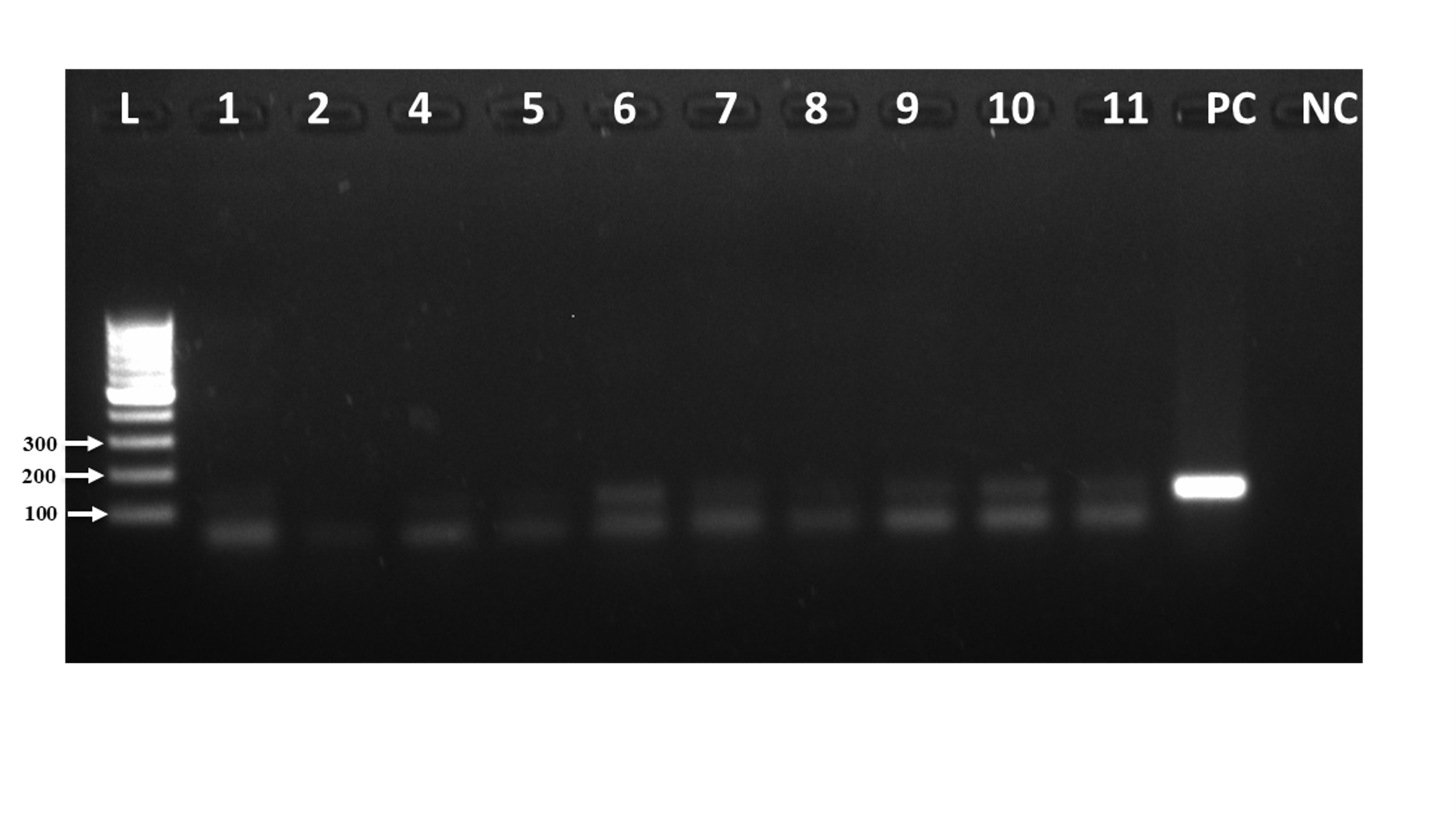
 **Supplementary figure S4:** A representative image of gel-electrophoresis of targeting *ctxA* gene. 1-11 are different samples. PC-positive control, NC-negative control, L=100 bp ladder. An expected band size 116bp showed positive result. Lower bands < 100bp in all samples is of primer dimers.
